# Predictive Simulations of Musculoskeletal Function and Jumping Performance in a Generalized Bird

**DOI:** 10.1093/iob/obab006

**Published:** 2021-04-15

**Authors:** P J Bishop, A Falisse, F De Groote, J R Hutchinson

**Affiliations:** 1 Structure and Motion Laboratory, Department of Comparative Biomedical Sciences, Royal Veterinary College, Hatfield, UK; 2 Geosciences Program, Queensland Museum, Brisbane, Australia; 3 Museum of Comparative Zoology, Department of Organismic and Evolutionary Biology, Harvard University, Cambridge, MA 02138, USA; 4 Department of Movement Sciences, KU Leuven, Leuven, Belgium; 5 Department of Bioengineering, Stanford University, Stanford, CA 94305, USA

## Abstract

Jumping is a common, but demanding, behavior that many animals employ during everyday activity. In contrast to jump-specialists such as anurans and some primates, jumping biomechanics and the factors that influence performance remains little studied for generalized species that lack marked adaptations for jumping. Computational biomechanical modeling approaches offer a way of addressing this in a rigorous, mechanistic fashion. Here, optimal control theory and musculoskeletal modeling are integrated to generate predictive simulations of maximal height jumping in a small ground-dwelling bird, a tinamou. A three-dimensional musculoskeletal model with 36 actuators per leg is used, and direct collocation is employed to formulate a rapidly solvable optimal control problem involving both liftoff and landing phases. The resulting simulation raises the whole-body center of mass to over double its standing height, and key aspects of the simulated behavior qualitatively replicate empirical observations for other jumping birds. However, quantitative performance is lower, with reduced ground forces, jump heights, and muscle–tendon power. A pronounced countermovement maneuver is used during launch. The use of a countermovement is demonstrated to be critical to the achievement of greater jump heights, and this phenomenon may only need to exploit physical principles alone to be successful; amplification of muscle performance may not necessarily be a proximate reason for the use of this maneuver. Increasing muscle strength or contractile velocity above nominal values greatly improves jump performance, and interestingly has the greatest effect on more distal limb extensor muscles (i.e., those of the ankle), suggesting that the distal limb may be a critical link for jumping behavior. These results warrant a re-evaluation of previous inferences of jumping ability in some extinct species with foreshortened distal limb segments, such as dromaeosaurid dinosaurs.

**Simulations prédictives de la fonction musculo-squelettique et des performances de saut chez un oiseau généralisé** Sauter est un comportement commun, mais exigeant, que de nombreux animaux utilisent au cours de leurs activités quotidiennes. Contrairement aux spécialistes du saut tels que les anoures et certains primates, la biomécanique du saut et les facteurs qui influencent la performance restent peu étudiés pour les espèces généralisées qui n’ont pas d’adaptations marquées pour le saut. Les approches de modélisation biomécanique computationnelle offrent un moyen d’aborder cette question de manière rigoureuse et mécaniste. Ici, la théorie du contrôle optimal et la modélisation musculo-squelettique sont intégrées pour générer des simulations prédictives du saut en hauteur maximal chez un petit oiseau terrestre, le tinamou. Un modèle musculo-squelettique tridimensionnel avec 36 actionneurs par patte est utilisé, et une méthode numérique nommée “direct collocation” est employée pour formuler un problème de contrôle optimal rapidement résoluble impliquant les phases de décollage et d’atterrissage. La simulation qui en résulte élève le centre de masse du corps entier à plus du double de sa hauteur debout, et les aspects clés du comportement simulé reproduisent qualitativement les observations empiriques d’autres oiseaux sauteurs. Cependant, les performances quantitatives sont moindres, avec une réduction des forces au sol, des hauteurs de saut et de la puissance musculo-tendineuse. Une manœuvre de contre-mouvement prononcée est utilisée pendant le lancement. Il a été démontré que l’utilisation d’un contre-mouvement est essentielle à l’obtention de hauteurs de saut plus importantes, et il se peut que ce phénomène doive exploiter uniquement des principes physiques pour réussir; l’amplification de la performance musculaire n’est pas nécessairement une raison immédiate de l’utilisation de cette manœuvre. L’augmentation de la force musculaire ou de la vitesse de contraction au-dessus des valeurs nominales améliore grandement la performance de saut et, fait intéressant, a le plus grand effet sur les muscles extenseurs des membres plus distaux (c'est-à-dire ceux de la cheville), ce qui suggère que le membre distal peut être un lien critique pour le comportement de saut. Ces résultats justifient une réévaluation des déductions précédentes de la capacité de sauter chez certaines espèces éteintes avec des segments de membres distaux raccourcis, comme les dinosaures droméosauridés.

**Voorspellende simulaties van musculoskeletale functie en springprestaties bij een gegeneraliseerde vogel** Springen is een veel voorkomend, maar veeleisend, gedrag dat veel dieren toepassen tijdens hun dagelijkse bezigheden. In tegenstelling tot de springspecialisten zoals de anura en sommige primaten, is de biomechanica van het springen en de factoren die de prestaties beïnvloeden nog weinig bestudeerd voor algemene soorten die geen uitgesproken adaptaties voor het springen hebben. Computationele biomechanische modelbenaderingen bieden een manier om dit op een rigoureuze, mechanistische manier aan te pakken. Hier worden optimale controle theorie en musculoskeletale modellering geïntegreerd om voorspellende simulaties te genereren van maximale hoogtesprong bij een kleine grondbewonende vogel, een tinamou. Een driedimensionaal musculoskeletaal model met 36 actuatoren per poot wordt gebruikt, en directe collocatie wordt toegepast om een snel oplosbaar optimaal controleprobleem te formuleren dat zowel de opstijg-als de landingsfase omvat. De resulterende simulatie verhoogt het lichaamszwaartepunt tot meer dan het dubbele van de stahoogte, en belangrijke aspecten van het gesimuleerde gedrag komen kwalitatief overeen met empirische waarnemingen voor andere springende vogels. De kwantitatieve prestaties zijn echter minder, met verminderde grondkrachten, spronghoogtes en spierpeeskracht. Tijdens de lancering wordt een uitgesproken tegenbewegingsmanoeuvre gebruikt. Aangetoond is dat het gebruik van een tegenbeweging van cruciaal belang is voor het bereiken van grotere spronghoogten, en dit fenomeen hoeft alleen op fysische principes te berusten om succesvol te zijn; versterking van de spierprestaties hoeft niet noodzakelijk een proximate reden te zijn voor het gebruik van deze manoeuvre. Het verhogen van de spierkracht of van de contractiesnelheid boven de nominale waarden verbetert de sprongprestatie aanzienlijk, en heeft interessant genoeg het grootste effect op de meer distale extensoren van de ledematen (d.w.z. die van de enkel), wat suggereert dat de distale ledematen een kritieke schakel kunnen zijn voor het springgedrag. Deze resultaten rechtvaardigen een herevaluatie van eerdere conclusies over springvermogen bij sommige uitgestorven soorten met voorgekorte distale ledematen, zoals dromaeosauride dinosauriërs.

**Prädiktive Simulationen der muskuloskelettalen Funktion und Sprungleistung bei einem generalisierten Vogel** Springen ist ein übliches jedoch anstrengendes Verhalten, das viele Tiere bei ihren täglichen Aktivitäten einsetzen. Im Gegensatz zu Springspezialisten, wie Fröschen und einigen Primaten, sind bei allgemeinen Arten, welche keine ausgeprägten Anpassung für Sprungverhalten aufweisen, die Biomechanik beim Springen und die Faktoren, welche die Leistungsfähigkeit beeinflussen, noch wenig untersucht. Computergestützte biomechanische Modellierungsverfahren bieten hier eine Möglichkeit, dies in einer gründlichen, mechanistischen Weise anzugehen. In dieser Arbeit werden die optimale Steuerungstheorie und Muskel-Skelett-Modellierung zusammen eingesetzt, um die maximale Sprunghöhe eines kleinen bodenlebenden Vogels, eines Perlsteisshuhns, zu simulieren und zu prognostizieren. Es wird ein dreidimensionales Muskel-Skelett-Modell mit 36 Aktuatoren pro Bein verwendet, und durch direkte Kollokation wird ein schnell lösbares optimales Steuerungsproblem formuliert, das sowohl die Abstoss- als auch die Landephase umfasst. Die daraus folgende Simulation bringt den Ganzkörperschwerpunkt auf mehr als das Doppelte seiner Standhöhe und entscheidende Aspekte des simulierten Verhaltens entsprechen qualitativ empirischen Beobachtungen für andere springende Vögel. Allerdings ist die quantitative Leistungsfähigkeit geringer, mit reduzierten Bodenkräften, Sprunghöhen und Muskel-Sehnen-Kräften. Beim Abstossen wird ein ausgeprägtes Gegenbewegungsmanöver durchgeführt. Die Durchführung einer Gegenbewegung ist nachweislich entscheidend für das Erreichen grösserer Sprunghöhen, wobei dieses Phänomen möglicherweise nur physikalische Prinzipien auszuschöpfen braucht, um erfolgreich zu sein. Die Verstärkung der Muskelleistung ist daher möglicherweise nicht zwingend ein unmittelbarer Grund für die Verwendung dieses Manövers. Eine Erhöhung der Muskelkraft oder der Kontraktionsgeschwindigkeit über die Nominalwerte hinaus führt zu einer erheblichen Zunahme der Sprungleistung und hat interessanterweise den grössten Effekt bei den weiter distal gelegenen Streckmuskeln der Beine (d.h. bei denjenigen des Sprunggelenks), was darauf hindeutet, dass die distale Gliedmasse ein entscheidendes Element für das Sprungverhalten sein könnte. Diese Ergebnisse geben Anlass zur Überprüfung früherer Schlussfolgerungen hinsichtlich der Sprungfähigkeit einiger ausgestorbener Arten mit verkürzten distalen Gliedmassen, wie beispielsweise bei dromaeosauriden Dinosauriern.

## Introduction

Many terrestrial animals are capable of jumping, and many frequently jump as part of their normal daily activities. Jumping is probably the most strenuous behavior that many animals regularly perform, with the rapid production of high forces and accelerations, over a wide range of limb or body angles, placing high demands on the musculoskeletal system (Biewener and Blickhan 1988; Demes et al. 1995; Henry et al. 2005; Alexander 1974). Relatively few species use steady-state jumping as a mode of locomotion (e.g., kangaroos, here termed “hopping”), but many use non-steady-state or explosive jumping for a variety of reasons, including negotiating complex environments, avoiding predators, or catching prey (Emerson 1978, 1985; Biewener and Blickhan 1988; Jackson and Willey 1994; Sunquist and Sunquist 2002; Toro et al. 2004; McGowan et al. 2005; Göttingen 2007; Hawlena et al. 2011). Jumping also has relevance in paleontology and evolutionary biology. Jumping has often been inferred as an integral component of the predatory strategies of various extinct carnivores (Paul 1988; Manning et al. 2006; Prothero 2016), and it may have also played a role in the evolutionary origin of novel behaviors (e.g., powered flight in birds; Earls 2000; Chatterjee and Templin 2012; Dececchi et al. 2016) or major clades (e.g., anurans and primates; Szalay and Dagosto 1980; Shubin and Jenkins 1995; Reilly and Jorgensen 2011; Boyer et al. 2017).

Experimental investigation of jumping biomechanics and performance in vertebrates has largely focused on humans, mostly in the context of sport (e.g., Hay 1993), or on jump-adapted species, mainly anurans and primates (e.g., Demes et al. 1995, 1996; Peplowski and Marsh 1997; Aerts 1998; Azizi and Roberts 2010; Legreneur et al. 2010; Astley and Roberts 2014; Bobbert et al. 2014; Porro et al. 2017). These species typically possess specialized (apomorphic) musculoskeletal anatomies that help improve jumping performance, well-known examples of which include the sacro-iliac joint (Jenkins and Shubin 1998) and plantaris “catapult” mechanism (Astley and Roberts 2012) of anurans and the proximal tarsus of prosimians (Boyer et al. 2013). However, relatively little work has been conducted on species that lack distinct musculoskeletal specializations for jumping, yet which nevertheless use jumping in daily activity (e.g., Zajac et al. 1981; Zajac 1985; Earls 2000; Harris and Steudel 2002; Toro et al. 2004; Henry et al. 2005; Alexander 1974). This makes it difficult to derive general principles about jumping capabilities that apply across broad groups of phylogenetically or morphologically disparate species, beyond those grounded in ballistics or gross anatomy, such as longer legs or greater limb muscle mass (Emerson 1985; Demes et al. 1998; Toro et al. 2004). The derivation of more precise principles is additionally complicated by the fact that experimental investigation of *in vivo* function is usually limited in the scale or scope of what can practically be measured in a given system, and these constraints are typically magnified by the practical difficulties of working with live non-human animals. Furthermore, with the exception of humans, it is difficult to experimentally elicit an organism’s true maximal performance in a given behavior, such as maximal jump height (Astley et al. 2013).

In light of these difficulties, computational modeling of the musculoskeletal system can provide an additional avenue by which to better understand the biomechanics of jumping, from neuromuscular control through to anatomical and mechanical constraints on performance (Bobbert 2013; Seth et al. 2018). Grounded in well-understood physical and biological principles, this approach can provide unique insight on various aspects of musculoskeletal function in a quantitative and rigorous fashion, that otherwise would be difficult (if not impossible) to study. For instance, practical or ethical considerations may limit the number of muscles (or other tissues) able to be investigated in any single individual or behavior, and some species are more difficult to obtain or less amenable to work with (e.g., are dangerous to handle, or are less “cooperative”). Computational models allow for the full complexity of the anatomical system involved (e.g., a limb, which may comprise upwards of 35 muscles) to be captured and analyzed in a mechanistic manner, in any species for which anatomical data are available. Musculoskeletal modeling, therefore, forms a valuable complement to experimental studies in comparative biomechanical enquiry, that can further advance understanding of jumping biomechanics in animals, particularly for species without readily identifiable skeletal adaptations for jumping or for which jumping behavior is unobserved (including extinct species).

One approach to computational musculoskeletal modeling that can prove particularly useful is predictive simulations of a model’s behavior forward through time. This allows different aspects of the system (e.g., muscle strength, limb dimensions, joint kinematics, and inertial properties) to be individually varied and their resultant effect on system behavior to be rigorously quantified (Alexander 1995; Moore et al. 2019), something not possible with *in vivo* experiments. One form of predictive simulation is to use experimental data, such as kinematics or joint moments, to drive the simulation forward, while varying other aspects of the system in a controlled fashion (e.g., Kargo et al. 2002; Parslew et al. 2018; Richards and Porro 2018; Richards et al. 2018; Nyakatura et al. 2019). These approaches have provided key insight into aspects such as stability, control strategies, and kinematic coupling, but are still to some extent bound to prior experimental data. An alternative approach is the application of optimal control methods to simulating model behavior (Anderson and Pandy 1999a; Ackerman and van den Bogert 2010; Umberger and Miller 2017). Using mathematical optimization of the system controls themselves (e.g., muscle excitations), simulations of behavior are generated independently of experimental data (and their inherent constraints) by seeking to maximize some physiologically relevant performance criterion. This permits exploration of “what if” questions, testing musculoskeletal function under any range of conditions *in silico*, and facilitating quantitative assessment of the relevance of anatomical or functional traits to performance in a given behavior.

Jumping is particularly amenable to this approach, as its performance criterion is fairly unambiguous and easily defined mathematically (Pandy et al. 1990): get as high vertically, or as far horizontally, as possible in a single maneuver. The application of optimal control methods for simulating maximal jump behaviors has a long history (Pandy et al. 1990; Anderson and Pandy 1993, 1999b; Spägele et al. 1999; Ashby and Delp 2006; Bobbert 2013; Ong et al. 2016; Porsa et al. 2016) and has provided important insight into the factors that influence jump performance in humans. However, the application of these methods to other species (extant or extinct) for any type of behavior remains in its infancy (Nagano et al. 2005; Sellers et al. 2005; Sellers and Manning 2007; Moore et al. 2013; Sellers et al. 2013a, 2013b, 2017; Sellers and Hirasaki 2018). Indeed, no study has applied optimal control methods to generating simulations of jumping performance in a non-human species, although Bobbert (2013) investigated a humanoid model scaled to various animal sizes. A key reason for this limited application is that optimal control simulations with sophisticated musculoskeletal models have historically been extremely computationally expensive to perform (due to the highly non-linear and stiff dynamic equations involved), typically requiring supercomputers. Recent application of alternate methods for solving optimal control problems (OCPs), particularly direct collocation methods, have greatly increased the speed at which complex OCPs can be solved, in some cases taking less than an hour to converge using standard computer hardware (Ackerman and van den Bogert 2010; van den Bogert et al. 2011; Lee and Umberger 2016; Porsa et al. 2016; Umberger and Miller 2017; Lin et al. 2018; Falisse et al. 2019a, 2019b, 2020). This, therefore, has the potential to greatly increase the pace of investigation into the biomechanics of jumping, and other behaviors, in diverse non-human species.

In this study, an approach to rapid OCP formulation and solving (Falisse et al. 2019a, 2019b) was combined with a high-fidelity musculoskeletal model (Bishop et al. 2021) to explore jumping mechanics and performance in the elegant-crested tinamou, *Eudromia elegans*. These small (∼500–700 g), ground-dwelling birds possess many musculoskeletal plesiomorphies insofar as the avian hindlimb is concerned (Cracraft 1974; McKitrick 1991; Worthy and Scofield 2012; Suzuki et al. 2014), and as such are representative of generalized species that lack obvious musculoskeletal specializations for increased jump performance (“generalized” being used as the antonym of “specialized”). There is no evidence that tinamous do not jump; they do run and fly, and presumably their launch from the ground to become airborne is predominantly hindlimb-driven, as in other ground-dwelling birds (e.g., Earls 2000; Henry et al. 2005). Hence, they can serve as an example bipedal system by which to understand how muscular and mechanical aspects can influence jumping performance in the absence of specialized anatomical adaptations, building on the seminal (but abstract) modeling work of Alexander (1995). Study of tinamous is also highly relevant for interpreting jumping ability, and its evolution, in the fossil record. Due to their phylogenetic heritage (as paleognaths), retention of small body size ancestral for Aves and Paleognathae (Worthy et al. 2017; Crouch and Clarke 2019) and retention of many hindlimb musculoskeletal plesiomorphies (Cracraft 1974; McKitrick 1991; Worthy and Scofield 2012; Suzuki et al. 2014), they presumably closely represent the ancestral avian body (hindlimb) plan among extant birds. Their propensity for ground-based life also implies that they are good at executing ground-based activities, such as jumping from (and landing on) firm substrates. Coupled with the fact that jumping is relevant to how birds transition from terrestrial substrates to aerial flight (Heppner and Anderson 1985; Bonser and Rayner 1996; Earls 2000; Provini et al. 2012; Provini and Abourachid 2018), the results of this study can therefore provide novel bearing for understanding the evolution of powered flight in birds, and potentially other vertebrate groups as well.

In introducing a novel computational approach to the field of comparative biomechanics, this study primarily sought to demonstrate the potential scope and power that rapid, optimal control predictive simulations bring to studies of musculoskeletal function and performance. It is hence of a broad, exploratory nature, without specific hypothesis-driven focus, although the general aim was to provide new insight into how jumping is achieved by species that lack musculoskeletal adaptations for jumping. Nonetheless, throughout the course of the study an interesting phenomenon was consistently recovered in simulations, the countermovement maneuver, whose relevance to jumping has been extensively debated (e.g., Anderson and Pandy 1993; Bobbert and Casius 2005; Henry et al. 2005). In addition to its overarching exploratory aim, this study therefore took on a secondary, subsidiary aim of further investigating this phenomenon as it relates to tinamou, helping to answer questions of the relative importance of muscle versus Newtonian mechanics in the achievement of jump performance.

## Materials and methods

Despite concerted efforts by the authors and colleagues to elicit vertical jumping in a controlled and measurable fashion, suitable experimental data were unable to be obtained for elegant-crested tinamou. Thus, in the current absence of experimental data, predictive simulations necessarily are the only means of quantitatively investigating jumping mechanics in this particular species. More broadly, when framed in a comparative context, such simulations form a powerful complement with other species for which experimental observations do exist, ultimately producing a more comprehensive understanding of avian jumping mechanics.

### Musculoskeletal model

A previously published three-dimensional musculoskeletal model of the tinamou was used (Bishop et al. 2021). This model was developed in OpenSim version 3.3 (Delp et al. 2007; Seth et al. 2018), has a mass of 545 g and comprises 9 body segments (including a rigid trunk) and 26 degrees of freedom (DOFs), with each hindlimb actuated by 36 muscle–tendon unit (MTU) actuators, covering all the important muscles of the leg (Supplementary Table S1). The previously derived parameters for each MTU were used here, as were the values for normalized tendon stiffness (*k*_T_ = 100) and activation and deactivation time constants (0.007 and 0.027 s, respectively). Foot–ground contact was modeled with a single contact sphere fixed to the digits segment. The physics of ground contact is exceedingly difficult to model accurately, especially with simplified representations such as that used here, but first principles suggest that contact parameters ought to vary with size. In the absence of any data to the contrary, the null hypothesis of isometry was assumed here; thus, contact stiffness was set to 250,000 N/m and dissipation was set to 0.0387 N·s/m, assuming isometric scaling with body mass with respect to a previously published human model (Lin and Pandy 2017; Falisse et al. 2019b). In contrast to the original model, abduction–adduction and long-axis rotation DOFs at the knee and ankle joints were omitted in this study (i.e., fixed at the reference pose of 0°), because these DOFs play a secondary role in limb kinematics and are mostly controlled by passive forces (Bishop et al. 2021), thus making the OCP more tractable. Abduction–adduction and long-axis rotation were retained at the hip joint, such that the revised model possessed 18 DOFs in total.

To facilitate rapid solving of the OCP, the optimization approach leveraged algorithmic differentiation (Falisse et al. 2019a), and all components of the musculoskeletal model were represented with twice continuously differentiable formulations. Variation in MTU length, velocity, and moment arms with respect to joint angles and velocities was represented with polynomial functions (Falisse et al. 2019a). Polynomials were fitted to the output from OpenSim’s MuscleAnalysis tool applied to 5000 randomly varying limb postures. Ultimately, all MTUs were represented using third- to fifth-order polynomials, with root mean square error of length <0.5 mm for almost all muscles (range 0–1.68 mm), and maximal root mean square error of moment arm <1 mm for almost all muscles (range 0–1.25 mm), compared with the original model.

### Standing posture

The jumping simulations in this study all began (and finished) in a bilaterally symmetrical, statically stable standing posture. The posture was derived firstly as an approximation of the midstance posture of walking (Bishop et al. 2021), but with knee and ankle abduction–adduction and long-axis rotation kept at zero, and hip abduction and rotation were modified until the center of foot–ground contact lay approximately underneath the whole-body center of mass (COM), and an appropriate mediolateral separation of right and left digits was present (i.e., toes from each foot did not step on each other). This first approximation was then optimized by adjusting pelvic height and limb angles so as to achieve perfect static balance, but which deviated from those in the initial posture to the minimal degree necessary (at most 5°). This was accomplished by transcribing the rigid body dynamics component of the musculoskeletal model to an algorithmically differentiable OpenSim C++ source file (Sherman et al. 2011; Falisse et al. 2019b), which was called as an external function within a custom MATLAB script version 9.5 (MathWorks, Natick, MA, USA; see code in Supplementary Material). The optimization problem was formulated using CasADi version 3.4.5 (Andersson et al. 2019) and solved with the interior-point solver IPOPT version 3.12.3 (Wächter and Biegler 2006).

### Predictive simulation

#### General overview of movement

The primary objective for jumping was defined here as maximizing vertical height of the whole-body COM, although there are other ways that jump performance could be characterized, depending on which is most ecologically relevant (Toro et al. 2004), and include maximizing horizontal distance, flight phase duration, or jump speed. To make the OCP more tractable, bilateral symmetry was imposed on the simulation so only the behavior of one leg had to be considered, and pelvic motion was constrained to the sagittal plane. This brought the total number of freely variable DOFs to 9 (pelvis pitch, and vertical and horizontal translations; hip flexion–extension, abduction–adduction, and long-axis rotation; knee, ankle, and metatarsophalangeal [MTP] angles) and the number of MTUs to 36. In contrast to many previous studies of predictive simulations of jumping, the present simulations included both liftoff and landing phases; just as stability during the liftoff will influence jump performance (Parslew et al. 2018), the ability to safely land will presumably also be important. Wing movement was not incorporated in the simulations (remaining part of the rigid trunk segment), with the hindlimbs providing all the force. Since this study is focused on the mechanics of hindlimb-driven jumping, this was deemed a suitable simplification, removing any potential confounding effect of the wings. Furthermore, studies of bird jumping or take-off for flight have shown that wing downstroke commences only toward the end of substrate contact or indeed after the feet have lost contact with the substrate (Heppner and Anderson 1985; Bonser and Rayner 1996; Earls 2000; Henry et al. 2005; Provini et al. 2012; Provini and Abourachid 2018), indicating that the hindlimbs provide most or all of the work required to become airborne.

#### OCP

System dynamic equations were represented using algorithmically differentiable implicit formulations. In the skeletal (rigid body) dynamics the states are generalized coordinates and velocities, and additional “slack” controls, which are the time derivatives of the generalized velocities (accelerations), were introduced; the non-linear dynamic equations were then imposed in the OCP as algebraic constraints in their implicit form. As part of this representation, foot–ground contact was modeled with a smoothed implementation of OpenSim’s Hunt–Crossley formulation (Sherman et al. 2011; Falisse et al. 2019a, 2019b). Excitation–activation and activation–contraction dynamics of the MTUs were implemented with the models of De Groote et al. (2016, 2009), where the states are muscle activations and tendon forces. Here, a change of variables (De Groote et al. 2009) meant that slack controls are the time derivatives of both muscle activations and tendon forces. The real controls of muscle excitations are back-calculated from the solution (they are not involved in its determination), and are uniquely determined by the activations and their time derivatives. The OCP was posed thus (see code in Supplementary Material): find the time-varying states **x**(*t*) and controls **u**(*t*) over the simulation duration *t*_0_ to *t*_final_ that minimized the objective function

(1) $$J=\int_{t_{0}}^{t_{\text{final}}}\left(\begin{array}{c} w_{1}\sum_{m=1}^{M}a_{m}^{2}+\\ w_{2}\sum_{l=1}^{L}T_{l}^{2}+\\ w_{3,\;\text{pelvis}}\sum_{p=1}^{P}{\ddot{q}}_{p}^{2}+w_{3,\;\text{leg}}\sum_{l=1}^{L}{\ddot{q}}_{l}^{2}+\\ w_{4}\left(\sum_{m=1}^{M}{\frac{da}{dt}}_{m}^{2}+\sum_{m=1}^{M}{\frac{dF_{T}}{dt}}_{m}^{2}\right) \end{array}\right)dt\;-w_{5}\cdot h_{\text{COM}},$$

subject to bounds on the states and controls

(2) $$x_{\min}\leq x(t)\leq x_{\max},$$

(3) $$u_{\min}\leq u(t)\leq u_{\max},$$

system dynamic equations

(4) $$f(x(t),\dot{x}(t),u(t))=0,$$

and path constraints on system behavior

(5) $$\Phi (x(t),\dot{x}(t),u(t),t)=0,$$

(6) $$\Psi (x(t),\dot{x}(t),u(t),t)\leq 0.$$

The objective function aimed to minimize five terms:

1. The sum of squared muscle activations (*a_m_*) across all *M* muscles (= 36 in the current model), integrated across the simulation. As explosive jumping at this spatial scale is likely limited by muscle power generation (Bennet-Clark 1977; Henry et al. 2005; James et al. 2007; Sutton et al. 2019), rather than muscle fatigue, a low weighting term was used (*w*_1_ = 0.01). This allowed for maximal muscle recruitment in generating high liftoff (and landing) forces, while keeping the OCP better conditioned than if such a term was not included.
2. The sum of squared passive joint moments (*T_l_*) across all *L* DOFs in the leg (= 6 in the current model) integrated across the simulation. In life, soft tissues (e.g., ligaments) will contribute to resisting extreme joint angles by providing passive restorative moments. In the absence of empirical data, passive moments were programmatically implemented as a double-exponential function of joint angle (Yoon and Mansour 1982; Silder et al. 2007); a single function was applied to each limb DOF by scaling to the respective bounds such that restorative moments started to be encountered when a given joint moved to within ∼15° of its upper or lower bound. A modest weighting term (*w*_2_ = 0.1) was used to discourage over-reliance of the model on passive moments in generating propulsive forces.
3. The sum of squared pelvis (${\ddot{q}}_{p}$) and leg (${\ddot{q}}_{l}$) coordinate accelerations across all *P* and *L* DOFs in the pelvis and leg, respectively (= 3 and 6 in the current model, due to bilateral symmetry), integrated across the simulation. This encourages the use of smoother model kinematics. The weightings used for all leg DOFs were set at *w*_3, leg_ = 0.1, but a markedly higher weighing was used for the pelvic DOFs (*w*_3, pelvis_ = 10). Preliminary tests demonstrated the necessity for using a higher weighting on pelvic DOFs so as to produce smoother, more realistic pelvic (and in turn trunk) movements.
4. The sum of squared time derivatives of muscle activations (d*a*/d*t*) and tendon forces (d*F*_T_/d*t*) across all *M* muscles, integrated across the simulation. The inclusion of this term, with a very low weight (*w*_4_ = 0.001), was to improve numerical conditioning of the OCP, avoiding situations for which these slack controls are not uniquely defined by optimality conditions (Falisse et al. 2019b).
5. The negative of the height reached by the whole-body COM (*h*_COM_) at the mid-point of the simulation, corresponding to the top of the flight (airborne) phase. A nominal weighting of *w*_5_ = 5 was used, but this was varied in sensitivity analyses.

The bounds on the leg DOF generalized coordinates were set based on the range of motion observed for each joint, determined by bone-on-bone collision in the musculoskeletal model (Bishop et al. 2021). The bounds on pelvic (trunk) pitch were set as [−15°, 45°], so as to allow reasonable flexibility while still keeping the head facing forward. Bounds on the generalized velocities and accelerations of all DOFs were set wide enough to allow for rapid movements. All other state (muscle activations and normalized tendon forces) and control (time derivatives of muscle activation and normalized tendon force) bounds were set in accordance with Falisse et al. (2019b). A variety of equality and inequality path constraints were imposed to guide system behavior, including: muscle excitation–activation and activation–contraction dynamics; zero residuals at the ground–pelvis joint (i.e., dynamic consistency); minimal mediolateral separation of the feet of 2 cm so that they did not touch or interpenetrate; a vertical whole-body COM velocity of zero at the mid-point of the simulation, defining the top of the flight path; and net moment balance between MTU forces, passive moments, external joint moments, and light joint damping (*d* = 0.0002 N·m·s/rad, applied to improve simulation smoothness) at each limb DOF. Additionally, the model was constrained to start and finish the simulation in the standing posture, but allowing for fore–aft horizontal displacement between the two to occur (i.e., pure vertical jumping was not enforced), and muscle states were constrained to be the same at the start and end of the simulation.

The above OCP, which is of infinite dimensionality, was transcribed to a non-linear program of finite dimension via direct collocation (Betts 2010) in CasADi. It was discretized across 100 evenly spaced mesh intervals for both liftoff and landing phases (200 intervals in total), and state continuity was enforced at each transitional mesh point. In the nominal simulation, jumping and landing phases were prescribed the same duration of 1.0 s (i.e., *t*_final_ = 2.0 s), although different durations for each could be prescribed. A “cold start” initial guess was used, comprising the static standing posture across the entire simulation duration; thus, no prior empirical knowledge of avian jumping behavior was provided to the simulation. To improve numerical conditioning, optimization variables were scaled as per Falisse et al. (2019b), although one difference was that the scaling factor used for d*a*/d*t* was increased in this study (set to 400), because of the rapid movements used in the simulated behavior. IPOPT was then used to solve the non-linear program, run on a standard 2.4 GHz processor. In addition to analyzing gross kinematic and kinetic aspects of the jump as a whole and the individual MTUs, muscle fiber and tendon work were calculated. This was achieved through integrating instantaneous power (product of force and velocity) with respect to time across the simulation, treating positive and negative powers separately to derive total positive and negative work.

### Sensitivity analyses

Following the solution of the nominal OCP defined above, five aspects of the OCP were modified in a series of one-at-a-time sensitivity analyses:

1. Maximal isometric force (*F*_max_) of MTUs was doubled for all MTUs. Prior simulation studies of strenuous behaviors in humans have sometimes found necessity to use increased values of *F*_max_ (or equivalently, maximum stress; e.g., Gerritsen et al. 1998; Miller et al. 2012; Arnold et al. 2013; Rajagopal et al. 2016), highlighting potential error in the anatomically-derived estimates of muscle strength. This sensitivity analysis thus explored the consequence of this potential error, but more broadly explored the relevance of muscle strength to jump performance; it would be expected that increased MTU strength would increase jump height. To provide further insight into how propulsive forces generated throughout the limb may contribute to jumping performance, MTU strength was also selectively increased (×2) or decreased (×0.75) for the extensor muscles of just the hip, knee, or ankle (seven analyses in total). The superscripts in Supplementary Table S1 denote which muscles were modified in each analysis.
2. Maximal muscle contraction velocity (*v*_max_) was doubled from 10 to 20 optimal fiber lengths (*ℓ*_o_) per second for all MTUs. A single uniform value for *v*_max_ was used in the nominal simulation, but it is possible that some muscles may have had higher values, and hence this sensitivity analysis sought to explore the general consequence of this. Since muscle power output may set a limit to jumping performance (Bennet-Clark 1977; Henry et al. 2005; James et al. 2007; Sutton et al. 2019), and jump-specialist species may have a higher proportion of fast-twitch muscle fibers (Lutz et al. 1998), it would be expected that increased *v*_max_ would increase jump height. This could occur either by allowing greater absolute muscle shortening velocities (translating into faster skeletal movement), by broadening the fiber force–velocity curve and enabling higher forces at the same absolute fiber velocity (McMahon 1984), or a combination of both. In a similar fashion to *F*_max_, *v*_max_ was also selectively increased for the extensor muscles of just the hip, knee, or ankle (four analyses in total).
3. Normalized tendon stiffness for all MTUs was both doubled (*k*_T_  = 200) and halved (*k*_T_ = 50). Since a generic value for absolute tendon stiffness (modulus) was assumed in the calculation of normalized stiffness (Bishop et al. 2021), this sensitivity analysis sought to explore the consequences of error introduced by such an assumption. Furthermore, one musculoskeletal mechanism inferred to play an important role in jump-specialist species is power modulation through elastic stretch and recoil of their in-series tendon (Aerts 1998; Astley and Roberts 2012; Astley and Roberts 2014), although this mechanism has also been hypothesized in generalized jumpers, too (Henry et al. 2005). Likewise, tendon stretch and recoil probably plays a role in power dissipation during rapid landing in at least some species (Konow et al. 2012; Konow and Roberts 2015).
4. Liftoff (*t*_lift_) and landing (*t*_land_) durations were increased (*t*_lift_ = *t*_land_ = 1.25 s) and decreased (*t*_lift_ = *t*_land_ = 0.75 and 0.5 s), for three analyses in total. The time available to appropriately position the body and coordinate the limbs during liftoff may influence total impulse, power output, or peak force production, and therefore a trade-off may exists between the speed of execution and jump height or distance (Toro et al. 2004). A similar argument can be made for the recovery of the starting position during landing.
5. Weighting on the jump height term in the objective function was increased and decreased (two analyses, *w*_5_ = 1 and 10). Whereas the previous four analyses sought to explore salient aspects that have clear potential biological relevance, this analysis intended to test the effect of a parameter that could be important to the OCP, yet whose value is difficult to quantify *a priori*.

A one-at-a-time approach to the 18 individual sensitivity analyses was used as it was more tractable than a full Monte Carlo or combinatorics sensitivity study, and the effect of each aspect on system behavior is clear. In addition to providing an assessment of the strengths and weaknesses of this study’s approach, such sensitivity analysis can further elucidate what aspects are more critical for jump performance, providing additional mechanistic insight not able to be gained from *in vivo* experiments.

### Exploring countermovements

Across all variants investigated, the kinematics of the resulting optimal solution consistently involved a countermovement maneuver (see the “Results” section). In the context of this study, a countermovement is defined as the use of a limb posture which is more crouched than that used during normal standing, and which is immediately preceded and succeeded by the use of less crouched postures (i.e., it is strictly a dynamic and transient behavioral phenomenon). To explore the mechanics of this maneuver and its relevance for vertical jump performance, additional jumping simulations were performed. First, to isolate the potential contributions of muscle–tendon mechanics and basic Newtonian mechanics, the nominal simulation was run with the model driven solely by torque actuators instead of MTUs (see Supplementary Material for details on implementation). Second, the torque-driven simulation was re-run, but set to start from the maximally crouched posture used in the nominal simulation (apogee of countermovement, taken to be the point where the whole-body COM was lowest), to ascertain whether further countermovement could be used. Lastly, both standing-start (nominal) and crouched-start simulations were re-run with an additional path constraint imposed, which forced the whole-body COM to monotonically increase in height over the jump, effectively minimizing the use of any countermovement strategy.

## Results

### Kinematics and kinetics

**Fig. 1:**
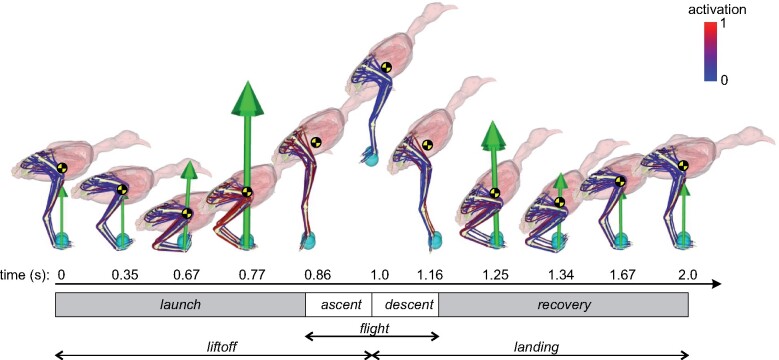
Graphical illustration of resulting kinematics, forces and muscle activations during the nominal jump simulation. Also shown is the location of the whole-body COM (yellow and black disc), and the different phases of the jump are indicated.

**Fig. 2:**
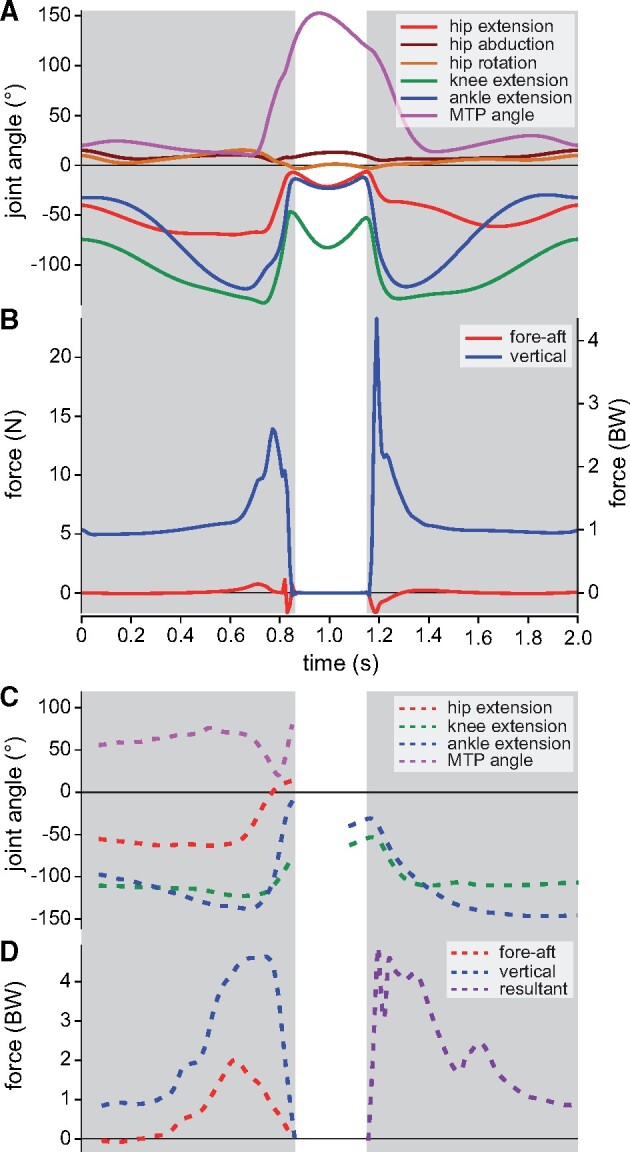
Time histories of nominal simulation limb kinematics (**A**) and GRFs (**B**, summed across both feet) compared with previous experimental data (**C, D**). Available experimental data for liftoff are for freely jumping guineafowl (Henry et al. 2005), and those for landing are for turkeys dropping 1.0 m (Konow and Roberts 2015). Note that comparison to these data is approximate only, since starting and stopping conditions (including posture) and timing are not completely equivalent between simulation and experiments.

The nominal OCP converged after 29 min and produced the kinematic and kinetic profiles reported in Figs. 1 and 2 and Supplementary Movie S1. For clarity, distinct phases throughout the jump sequence are recognized thus (Fig. 1): liftoff comprises launch and ascent, and landing comprises descent and recovery; moreover, ascent and descent collectively form the flight phase. Starting from a standing height of 0.133 m above the ground, the whole-body COM reached a height of 0.305 m at the top of the flight path; jumping was not purely vertical, with a net forward translation of 0.05 m from start to finish. A key feature of the launch phase was the execution of a strong countermovement prior to full limb extension and loss of ground contact, during which hindlimb flexion lowered the whole-body COM to less than half its standing height (0.052 m). A similar countermovement was also executed during the recovery phase of landing, although this was less extreme (COM height 0.078 m). Both countermovements were accomplished mostly through knee and ankle flexion–extension. The hindlimb remained relatively still and extended during the majority of the flight phase.

As far as can be discerned, hindlimb kinematics are mostly comparable to those reported previously for liftoff (Henry et al. 2005) and landing (Konow et al. 2012) sequences in galliform birds (Fig. 2A, C); strict quantitative comparison is largely not possible (see the “Discussion” section). However, one discrepancy is the strong dorsiflexion of the MTP joint during the countermovements. In qualitative terms, temporal patterns of the vertical and horizontal components of the ground reaction force (GRF) also compare well with those reported previously (Fig. 2B, D; Bonser and Rayner 1996; Earls 2000; Henry et al. 2005; Konow and Roberts 2015). During launch, vertical GRF temporarily drops below one body weight (BW) as countermovement progresses, and then increases sharply toward the end, peaking at 2.62 BW before liftoff, in association with a small forward-directed horizontal GRF. This pattern is effectively reversed during the recovery phase, although peak vertical GRF at landing is higher, at 4.33 BW. Forces change most quickly immediately before and after the flight phase. Although experimental data for jumping tinamous are lacking, jumping performance in the model is apparently less than what may be expected based on prior observations for guineafowl (Henry et al. 2005). In that study, the birds (∼1.42 kg) raised their whole-body COM by ∼0.8 m or four times standing COM height, and produced peak vertical GRFs in excess of 4 BW. Even if scaling effects are ignored, this is substantially better than the tinamou model’s performance.

### MTU behavior

**Fig. 3:**
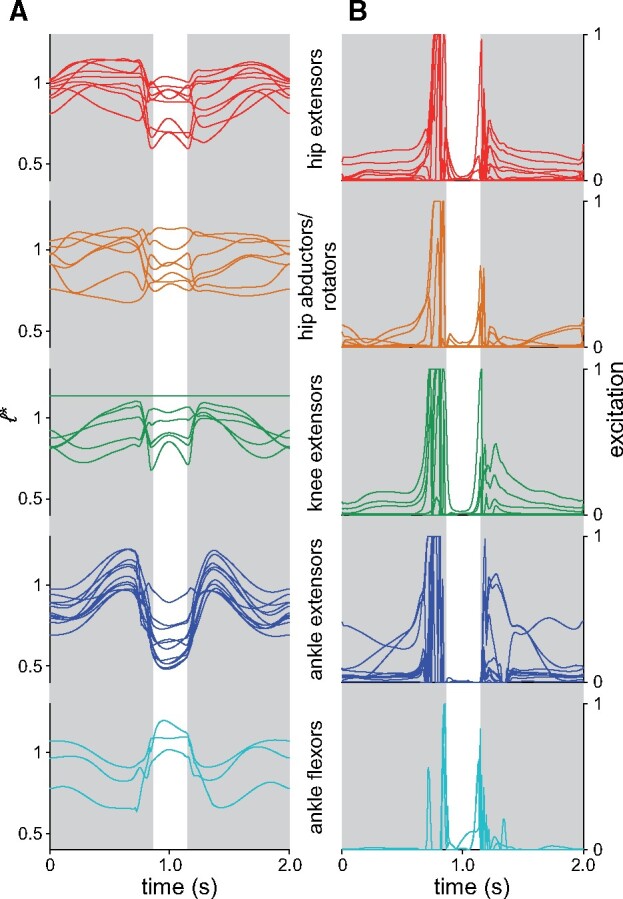
Time histories of normalized fiber lengths (**A**) and excitations (**B**) for each MTU in the nominal simulation parsed by major functional group. The femorotibialis lateralis as modeled does not undergo any appreciable length change across the knee’s range of motion (Bishop et al. 2021).

**Fig. 4:**
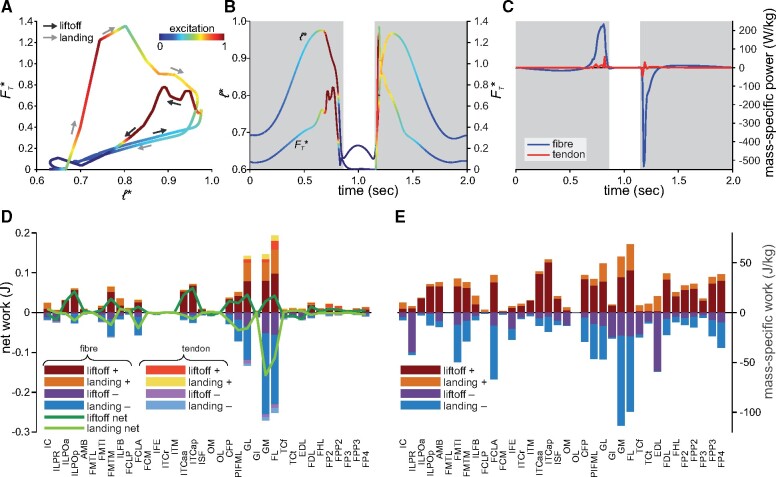
Energetic aspects of MTU behavior in the nominal simulation. (**A**) Exemplar work loop, plotted for the GM, comparing normalized tendon force (F_T_* = tendon force divided by *F*_max_) and normalized fiber length. (**B**) Time histories of normalized tendon force, normalized fiber length, and excitation for the GM. (**C**) Time histories of muscle mass-specific fiber and tendon power, summed across the extensor muscles analyzed previously for guineafowl by Henry et al. (2005). (**D**) Net positive and negative work for fibers and tendons in each MTU, partitioned between liftoff and landing phases; also plotted is the net fiber work during liftoff and landing. (**D**) Mass-specific fiber work in each MTU, partitioned between liftoff and landing phases. See Supplementary Table S1 for abbreviations.

Across all MTUs, fibers operated largely on the ascending limb or plateau of the active force–length curve, with normalized fiber lengths (ℓ* = *ℓ/ℓ*_o_) varying mostly between 0.5 and 1.2 (Fig. 3A). MTUs with high fiber pennation tended to show a discord between fiber and MTU velocities (Supplementary Fig. S1), indicating a decoupling of fiber length change from MTU length change; this served to keep fibers operating closer to the peak in the active force–length curve, improving force-producing capacity. Normalized fiber velocity for most muscles tended to remain lower (closer to being isometric) than that at which peak power is achieved (Supplementary Fig. S2), indicating that, in general, force production was favored over power production. Paralleling the temporal pattern in GRFs, MTU excitations rapidly increased toward the end of launch and at the beginning of recovery, but were very low during the intervening flight phase (Fig. 3B). Three-quarters of all MTUs were maximally recruited during launch, and many were also near-maximally recruited during initial recovery. During launch countermovement, the three strongest ankle extensors, the gastrocnemii lateralis (GL), et medialis (GM), and fibularis longus (FL), were recruited while their fibers were still lengthening, increasing fiber force through active stretch (Fig. 4A, B). These muscles, and other ankle extensors, subsequently underwent rapid, active contraction over the remainder of the launch.

Mass-specific fiber power, summed across all MTUs, shows the same qualitative pattern during the liftoff phase as reported for guineafowl (Henry et al. 2005), with low negative power during countermovement, followed by a shorter burst of high positive power, peaking just before the flight phase (Fig. 4C). Considering just the extensor muscles analyzed by Henry et al. (2005), mass-specific power during liftoff peaked at 234 W/kg, less than a third of peak power for guineafowl; a greater power (−536 W/kg) was briefly used in landing. Partitioning muscle fiber work into positive and negative contributions to the liftoff and landing phases (Fig. 4D) reveals that many MTUs illustrated a common pattern of high positive and low negative work during liftoff, and low positive and high negative work during landing. (Work due to damping effects and passive joint moments was negligible compared with the work required to raise the whole-body COM.) For many MTUs the net work produced (or in some cases, absorbed) during liftoff was markedly different from that absorbed (or in some cases, produced) during landing; landing was therefore not simply “liftoff played in reverse.” Large quantities of positive and negative work were produced and absorbed by key extensor muscles of the hip (iliotibialis lateralis pars postacetabularis [ILPO], flexor cruris lateralis pars accessoria [FCLA] and puboischiofemoralis medialis et lateralis [PIFML]), knee (femorotibialis medialis [FMTM]), and ankle (GL, GM, and FL). Normalizing for muscle mass, positive and negative fiber work frequently exceeded 20 J/kg in both liftoff and landing phases (Fig. 4E), but this is only about half of the mass-specific fiber work estimated for jumping guineafowl by Henry et al. (2005), and about a third of maximal mass-specific fiber work for a single contraction in frog muscle calculated by Peplowski and Marsh (1997).

Despite many MTUs exhibiting a stretch–shorten fiber trajectory during liftoff and landing, almost all of this was taken up in the fibers, with low tendon strain (<1.6%) across the simulation for all MTUs. Muscle mass-specific tendon power generally remained <20 W/kg (briefly peaking at 58 W/kg during launch), far lower than estimates of peak power for the GL tendon in landing turkeys (>1 kW/kg; Konow and Roberts 2015). Consequently, elastic potential energy storage and recovery in the tendons was minimal, with net positive or negative tendon work remaining <0.03 J for any MTU (Fig. 4D), and total positive tendon work in liftoff amounting to 0.096 J (10.3% of work required to raise the COM).

### Sensitivity analyses

**Fig. 5:**
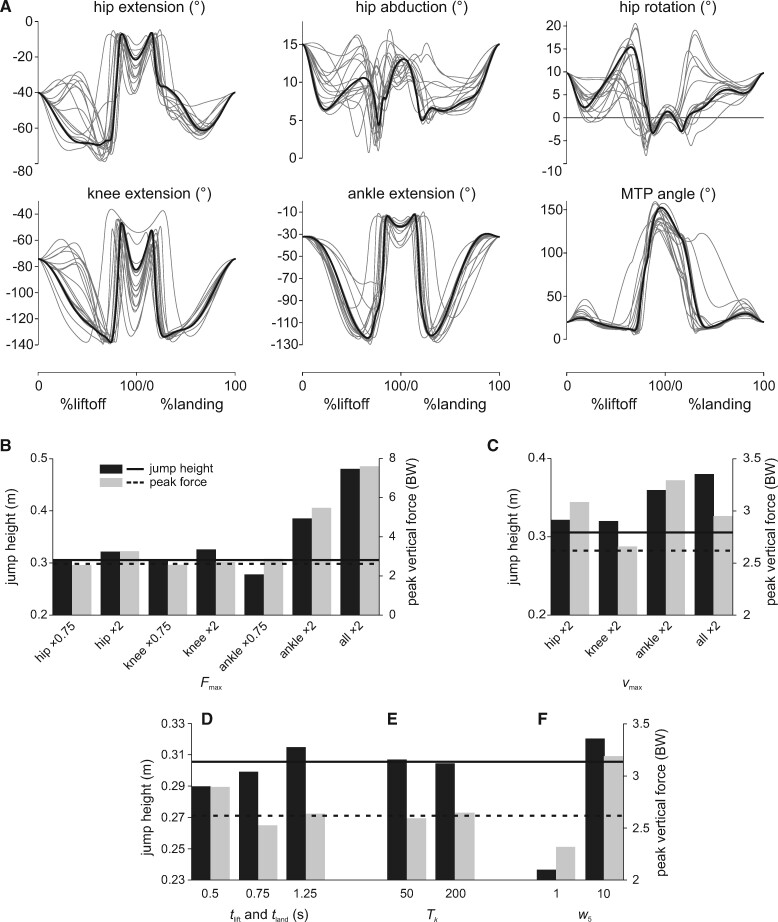
Results of sensitivity analyses. (**A**) Temporal trajectories of limb angles for all 18 variants (gray lines) compared with those of the nominal simulation (black line). For comparison the trajectories of all simulations are scaled to the same duration of liftoff and landing phases. (**B–F**) Effects on jump height and peak vertical GRF (summed across both feet), for alterations to muscle *F*_max_ (**B**), *v*_max_ (**C**), liftoff and landing phase durations (**D**), normalized tendon stiffness (**E**), and jump height weighting factor in the objective function (**F**). Horizontal lines denote the nominal simulation results. Note the differences in vertical axis scale between **B, C,** and **D–F.**

With one exception, limb kinematics in all 18 sensitivity analyses was highly similar to that in the nominal simulation, mostly being scaled by duration and timing (Fig. 5A and Supplementary Movie S2); higher jumps lead to longer duration flight phases interposing the launch and recovery sequences. Increasing *F*_max_ for all MTUs markedly increased jump height compared with the nominal simulation, with the whole-body COM reaching 0.48 m above the ground (Fig. 5B and Supplementary Movie S2), a 101% improvement in vertical COM displacement compared with the nominal simulation; peak vertical GRF during liftoff reached 7.6 BW, a 190% increase from the nominal simulation. Increasing *F*_max_ for the extensors of just one joint at a time had a similar effect, and the converse was largely true for decreasing *F*_max_. However, the influence on jump performance was most marked for the ankle joint, despite the hip extensors comprising a 51% greater proportion of limb muscle mass than the ankle extensors (Supplementary Table S1). Increasing *F*_max_ for all MTUs more than doubled peak total mass-specific fiber power for the extensors (516 W/kg), but this still fell short of estimates of 712–778 W/kg for jumping guineafowl (Henry et al. 2005). Mass-specific fiber work for many muscles exceeded 50 J/kg in liftoff or landing, and mass-specific tendon power peaked at 140 W/kg toward the end of liftoff, but net positive or negative tendon work was minimally increased (<0.05 J).

In a similar fashion to *F*_max_, increasing *v*_max_ also improved jump performance, although not by as much (Fig. 5C and Supplementary Movie S2), and this was also reflected by more modest increases in mass-specific fiber and tendon powers (379 and 72 W/kg, respectively). As normalized fiber velocities for most muscles tended to remain closer to 0 than in the nominal simulation (Supplementary Fig. S2), this implies that a least part of the improvement in jump height resulted from the exploitation of greater force-producing capabilities at about the same absolute fiber velocities, rather than just an increase in absolute fiber velocities. Paralleling the result for *F*_max_, improvements in jumping performance resulting from increases in *v*_max_ were most marked for extensors of the ankle.

Altering *k*_T_ or *t*_lift_ and *t*_land_ had far less of an impact on jump performance, with lower tendon stiffness and longer phase durations leading to slight increases in jump height (Fig. 5D, E and Supplementary Movie S2). The more compliant tendon simulation (*k*_T_ = 50) that improved jump performance involved a slightly lower mass-specific fiber power (211 W/kg) but a markedly higher mass-specific tendon power (peaking at 130 W/kg); not surprisingly, elastic energy storage was almost doubled, with total positive work during liftoff of 0.177 J (18.9% of work required to raise the COM). Fiber and tendon power and work in the long-duration simulation (*t*_lift_ = *t*_land_ = 1.25 s) were extremely similar to those in the nominal simulation. Unsurprisingly, higher and lower values for *w*_5_ resulted in higher and lower jump heights being achieved, respectively (Fig. 5F and Supplementary Movie S2); the kinematics for the higher weighting simulation were noticeably different from those of the nominal simulation and the observations of Henry et al. (2005), retrospectively supporting the nominal value used.

### Countermovement tests

**Fig. 6:**
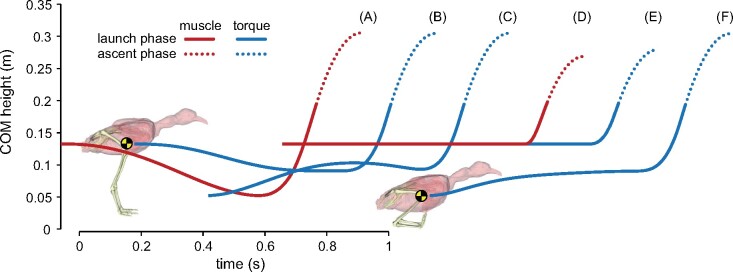
Exploration of the use of a countermovement on the trajectory of the whole-body COM (yellow and black disc). (**A**) Nominal muscle-driven simulation. (**B**) Nominal torque-driven simulation. (**C**) Torque-driven simulation starting from a crouched pose. (**D**) Muscle-driven simulation, constrained so that the COM height monotonically increases over the liftoff phase. (**E**) Torque-driven simulation, constrained so that the COM height monotonically increases over the liftoff phase. (**F**) Torque-driven simulation, starting from a crouched pose and constrained so that the COM height monotonically increases over the liftoff phase.

Owing to substantially fewer design variables and constraints, the OCPs involving a torque-driven model were substantially quicker to solve, often converging in less than 5 min. The nominal torque-driven simulation used slightly different kinematic trajectories compared with the nominal muscle-driven simulation, and experienced a net negative horizontal translation of 0.04 m (Supplementary Movie S3), but otherwise achieved the same whole-body COM height at the top of the flight phase (Fig. 6A, B). This torque-driven simulation also employed countermovements during launch and recovery phases, although neither was as pronounced as in the nominal muscle-driven simulation. When starting from the crouched pose, the torque-driven model did not lower its COM any further before executing the liftoff (effectively, a countermovement was not used), but still was able to raise its COM to the same height (Fig. 6C). Constraining the COM to monotonically increase in height over the liftoff phase substantially reduced jump height if the model started from a standing position (Fig. 6D, E), but it was unaffected if the model started from the maximally crouched position (Fig. 6F).

## Discussion

Using optimal control methods, this study sought to gain new insight into the mechanics of avian jumping, and to investigate what factors may influence jumping performance in “generalized” species that lack specialized anatomical adaptations for this behavior. In the nominal simulation, the tinamou model raised its whole-body COM >30 cm above the ground, more than double its standing height, which was greatly improved upon with increased muscle strength (*F*_max_) or, to a lesser degree, speed (*v*_max_). The present simulations are the first time that jumping has been simulated in a non-human species using optimal control methods and a high-fidelity musculoskeletal model, providing the foundations for future investigations targeted at better exploring musculoskeletal function and performance in diverse vertebrate species. This includes apomorphic anatomical systems or idiosyncratic behaviors, features which are unusual or restricted to a select group of species, that have evaded current understanding. Additionally, this study is one of few computational studies to have investigated both liftoff and landing together (Spägele et al. 1999; Ong et al. 2016). In comparison to the liftoff phase, landing remains disproportionately understudied, in any species, and for birds appears to have been investigated mostly in the context of descent from flight (e.g., Bonser and Rayner 1996; Provini et al. 2014). Yet landing is an integral component to many jumps, and the mechanics of this phase is therefore also of great biological relevance. The results of the present simulations indicate that landing is not necessarily just “liftoff played in reverse,” in terms of kinematics, kinetics, or muscle function, suggesting that differing constraints or selective pressures may act on the different phases of the jump. Indeed, landing potentially has its own unique aspects that require consideration, such as safety (e.g., reduction of tissue loading upon impact; Konow and Roberts 2015) and accuracy (e.g., jumping to a particular position in space), highlighting a rich avenue of future inquiry. Again, the computational approach employed here provides a foundation to quantitatively and mechanistically explore this important behavior.

### Countermovements

An important result obtained here is that, despite no encouragement or constraint to do so in the OCP (including the use of a “cold start” initial guess), the model consistently and spontaneously performed a countermovement before executing the liftoff (Fig. 1 and Supplementary Movie S1). A wide variety of animals employ this maneuver in jumping, including many birds (although it is by no means ubiquitous; Earls 2000; Tobalske et al. 2004; Henry et al. 2005; Provini et al. 2012; Provini and Abourachid 2018; Cox et al. 2020), and it has also been recovered in previous predictive simulations of human jumping (Pettersson et al. 2013; Ong et al. 2016). Countermovement jumps are well known to be higher than squat jumps in humans (Bobbert et al. 1996), and their widespread use among various species may be due to one or more factors. First, limb flexion during countermovement may increase extensor muscle forces through active stretching, enhancing propulsive force production later in the launch (Anderson and Pandy 1993; Bobbert and Casius 2005). Second, MTU power modulation may occur through slow tendon stretch followed by rapid recoil (Henry et al. 2005), analogous to the mechanism of tendon-mediated power modulation in jump-specialist species that do not typically employ countermovements (Aerts 1998; Roberts and Marsh 2003; Astley and Roberts 2012). It is probable that the first mechanism was involved to some extent in the tinamou simulations, since the GL, GM, and FL underwent active lengthening. By contrast, the very low tendon power and work in the present simulations suggest that the second mechanism did not play an important role (see also below).

The results of simulations using a torque-driven model (Fig. 6 and Supplementary Movie S3) indicate that a third, purely physics-based mechanism was also involved. A simulation driven solely by torque actuators also used a countermovement, and was able to raise the whole-body COM as high as the muscle-driven simulation; this implies that muscular mechanisms are not necessarily required, and that physics alone may explain the widespread use of a countermovement maneuver during jumping in diverse vertebrate species. Indeed, such a physics-based mechanism may be the principal means by which generalized species maximize their jumping performance. Further insight was gained from the results of simulations with the model either starting in a maximally crouched pose, or constrained to raise COM height in a monotonic fashion (Fig. 6C–E). These collectively demonstrated that if starting in a standing pose, a countermovement is needed to maximize jump height, whereas starting in a crouched pose obviates such a necessity. Thus, the underlying physical mechanism responsible for the use of countermovements is probably that increased limb flexion increases the vertical distance the COM can travel while limb work can be done on it, translating into a greater gravitational potential energy at the top of the flight phase (i.e., greater jump height). Equivalently, for the same net vertical distance traveled by the COM, a more crouched posture can increase the duration over which vertical propulsive forces can be applied on the substrate (before the feet leave the ground), allowing for greater vertical impulse to be generated (Gray 1968; Emerson 1985).

### Comparison to empirical observations

Experimental data on jumping were unable to be attained for tinamou: a key reason motivating this study. It remains unknown as to the exact sequence of kinematics or kinetics used by this species in executing such a maneuver, or what their maximum performance is. Recourse must therefore be made to previous experimental observations of jumping in other avian species. However, the absence of direct comparison is not considered to pose an insurmountable hindrance to gaining further insight on the underlying mechanics of avian jumping, because available experimental evidence for other species indicates no outward differences in how different birds jump from a terrestrial substrate, insofar as the hindlimbs are concerned (Heppner and Anderson 1985; Bonser and Rayner 1996; Earls 2000; Henry et al. 2005; Provini et al. 2012; Provini and Abourachid 2018). That is, except perhaps for scaling effects, there is no *a priori* reason to expect tinamous to jump differently to other species, at least in the qualitative sense of the time histories of kinematics and kinetics. The study of how avian jumping mechanics may scale with size or shape across extant species was beyond the scope of this study, but the mechanistic model of avian jumping developed here provides the requisite foundation for this to occur in the future. Scaling considerations aside, there are several issues that still limit direct, quantitative comparison of kinematics between the simulations and previously reported experimental data for other species, or even between prior experimental data for different species. Different studies use different conventions for reporting joint angles (many are also only two-dimensional, reducing accuracy), some studies only report data for a subset of all limb joints, and different studies (including the present one) involve different starting or finishing conditions. Species with different intralimb proportions will also inherently use different limb kinematics (Gatesy and Pollard 2011). Additionally, as noted in the “Introduction” section, it can be difficult to experimentally elicit certain behaviors in a controlled fashion for study, and can be even more difficult for maximum performance behaviors. These issues highlight the potential value that computational modeling and simulation studies can bring to investigations, by providing quantitative and mechanistic insight on aspects that are otherwise difficult or impossible to study (see also below).

Given the above remarks, the qualitatively similar temporal profiles for GRFs and limb kinematics between the present simulations and those reported for other species is encouraging (Bonser and Rayner 1996; Earls 2000; Henry et al. 2005; Konow and Roberts 2015), and lends credence to the simulations (“validity”; see below) as a whole. Particularly noteworthy is the spontaneous use of a countermovement maneuver, which for all species (as far as can be determined) is driven primarily by flexion of the knee and ankle joints. One marked discrepancy in kinematics was the non-realistic behavior of the MTP joint in the launch and recovery phases (Fig. 1 and Supplementary Movie S1). This is a consequence of the simplified representation of the pes and contact with the ground, the digits being modeled as a single rigid body with a single contact sphere rigidly fixed to it. The observed behavior of the MTP joint allowed for a smooth rolling of the sphere back and forth as the center of pressure shifted anteroposteriorly as required to accommodate movements in more proximal parts of the limb and body. A further non-physiological aspect of MTP behavior was its disposition at the moment of landing impact, with the digits straightened out and in line with the GRF (Supplementary Movie S1). This was likely due to the objective function in the OCP being focused on maximizing jump height, without factoring in safe tissue loading (e.g., joint contact forces).

The other main contrast between simulation results and previous experimental work is the apparently lower performance of the simulation in achieving height. Compared with a previous study of jumping guineafowl (Henry et al. 2005), the nominal tinamou simulation raised whole-body COM only about half as high (in proportional terms), produced ∼40% lower peak vertical GRFs and less than a third of peak mass-specific muscle fiber power. These differences in magnitude probably reflect, to at least some degree, genuine differences between tinamous and the guineafowl studied by Henry et al. (2005). For instance, tinamous have a lower proportion of body mass (14.1% across both limbs) invested in hindlimb musculature than guineafowl (22.7% across both limbs; Henry et al. 2005), which may explain lowered absolute performance (Emerson 1985; Demes et al. 1998; Toro et al. 2004), but does not explain the reduced fiber power. The peak fiber power reported for guineafowl, estimated by an inverse dynamics approach, was considerably higher than known isotonic power output for turkey limb muscle (Nelson et al. 2004) and led to the suggestion of elastic energy storage in, and power amplification by, tendons, particularly those of the ankle extensors. Elastic energy storage and power dissipation by ankle extensors has also been noted for turkeys in drop-landing maneuvers (Konow et al. 2012; Konow and Roberts 2015). Yet, in the tinamou simulations tendon stretch-and-recoil was minimal, with very low elastic energy storage and tendon power (Fig. 4).

The minimal stretching of tendons as found here likely stem from higher normalized tendon stiffness in tinamous, which was estimated from anatomical measurements of tendons in dissection (Bishop et al. 2021), although a generic value for modulus was used (Ker 1981). High normalized tendon stiffness is consistent with smaller animals generally having lower ratios of muscle physiological cross-sectional area compared with tendon cross-sectional area (Alexander et al. 1981; Bennett 1996; McGowan et al. 2008; Lamas et al. 2014), which in turn limits their ability to utilize tendon stretch-and-recoil in executing steady or unsteady movements (Biewener and Blickhan 1988; Moore et al. 2017). Changing tendon stiffness in the sensitivity analyses had only minimal effect on tendon stretch and tinamou jump performance, suggesting that tendon compliance and power amplification may not be important to tinamou jumping. Despite this, jump-specialist primates and anurans that are even smaller than tinamous probably employ tendon stretch-and-recoil in powering jumps (Aerts 1998; Roberts and Marsh 2003; Astley and Roberts 2012), implying that these species may have exceptionally compliant tendons for their body size, in relative if not absolute terms. Clearly, further empirical study of tendon properties and mechanics in a variety of both generalized and jump-specialist species is needed.

The apparently reduced jumping performance of the nominal simulation may also result in part from the underlying mathematical model of muscle force production. It is well-known that Hill-type models of activation–contraction dynamics have shortcomings in accounting for muscle lengthening (e.g., residual force enhancement), stretch-shortening or time-dependent effects, rapid changes in state or actin–titin interactions (Günther et al. 2007; Herzog 2014; Nishikawa 2016), at least some of which occur in liftoff and landing here. In order to better replicate observed performance, modeling studies of strenuous behavior in humans consequently often employ increased muscle strength, or use estimates derived directly from subject-specific strength measurements (Gerritsen et al. 1998; Anderson and Pandy 1999b; Miller et al. 2012; Arnold et al. 2013; Rajagopal et al. 2016). The doubling of *F*_max_ in the tinamou simulation substantially increased jump performance in terms of kinematics and kinetics, although mass-specific fiber power remained lower than that estimated for guineafowl. An alternative factor is that the current formulation of the OCP may not capture the entire set of objectives and constraints inherent to vertical jumping. As noted above, fiber operating velocities were generally lower than the speed that maximizes power, perhaps indicating that force production was favored over power production in the current simulations.

### On “validation”

Even though no experimental data for jumping in tinamou exist for direct (“gold standard”) comparison, the simulations were broadly successful in helping to achieve this study’s aims. Despite some probable deviations from empirical expectations noted above, it is important to recognize that the simulations were nonetheless able to spontaneously replicate key kinematic and kinetic observations made for other avian species, without any prior knowledge of how a bird should actually jump. This result raises the philosophical question of how much is required for a predictive simulation to be considered “validated” (i.e., sufficiently representative for the study’s purposes) especially in an extant species where numerous studies of related species with similar (and homologous) anatomies and functions already exist. Certainly the best case scenario would involve high quality, subject-specific kinematics, kinetics, electromyography, sonomicrometry, tissue property, and other data to test the accuracy of the simulations in estimating actual function and performance (e.g., Hutchinson 2012). However, it is argued here that such a case is the extreme end of what should be viewed as a *continuum of validation* (testing), rather than a binary perspective of “enough data (validated)” versus “not enough data (not validated).” The present study lies somewhere in the middle along this continuum, where limited data from other avian species are available for comparison: they provide indirect or qualitative tests of model validity (Henninger et al. 2010; Lund et al. 2012). These data are far better than none at all, and in tandem with the simulation results help further build a foundation for future exploration of many aspects of avian jumping mechanics. Approaching the issue of validation through the lens of diametrically opposite viewpoints as “validated”/“not validated” can obscure nuances that could otherwise provide a more comprehensive understanding of a particular topic; as in most other aspects of science, such false dichotomies ultimately impede progress (Hutchinson and Allen 2009). A more useful perspective may be to consider this continuum rather as one of “model evaluation” (Oreskes 1998; Nigg and Herzog 2007): does the model or simulation output have too poor of a match to any available independent data (or do sensitivity analyses raise substantial doubt) for it to be considered unreliable for addressing a given study’s aims? The plausibility of a given simulation obviously increases as more empirical data are favorably compared with it, but since all models are by definition “wrong,” a perfect match between simulation and empirical observation (even those of a gold standard) ought not to be expected. Indeed, sometimes the discord between theoretical modeling and experimental datasets can prove particularly informative; for example, investigating how the formulation of the objective function in the OCP affects the level of agreement between simulation and reality can provide insight into what real animals actually seek to prioritize *in vivo* when executing a given behavior (Nguyen et al. 2019; Zargham et al. 2019). Rather than being viewed independent of (or worse, in opposition to) one another, computational modeling and empirical studies can—and should—be reconciled as complementary approaches that can provide important reciprocal illumination (cf., Campione and Evans 2020; Wiseman et al. 2021); just as *in vivo* experimental studies can help verify the biorealism of *in silico* simulations, simulations can help identify those aspects of a system most in need of further empirical scrutiny, and can suggest new programs of experimental testing. An evolving dialog between theoretical and empirical approaches will therefore facilitate greater improvement in understanding of organismal biology.

### Jumping in non-specialized jumpers

In addition to providing a framework for computational estimation of jumping performance, this study provides further clarity on aspects that influence vertical jumping performance in generalized species that lack distinct anatomical specializations for jumping. Paralleling prior empirical and theoretical studies, increased muscle strength (or equivalently, increased muscle bulk) and maximum contractile speed improve jumping performance (Emerson 1978, 1985; Alexander 1995; Demes et al. 1998; Toro et al. 2004). However, the magnitude of these effects showed a proximal–distal gradient, with changes to strength or contraction speed for ankle extensors having the most marked influence (Fig. 5B, C), despite the ankle extensors comprising two-thirds the muscle mass of the hip extensors and only 27% more muscle mass than the knee extensors. This suggests that actuation of the distal limb is more important to jumping. Indeed, jump-proficient species tend to not only have longer limbs, but longer distal limb segments as well, especially the foot (Zug 1972; Emerson 1985; Boyer et al. 2013; Moore et al. 2015); theoretical modeling by Alexander (1995) also reached a similar conclusion. Thus, longer distal limb segments permit larger endpoint (foot) displacements relative to the body, prolonging foot–ground contact duration and increasing the applied impulse during launch, increasing vertical momentum.

An explicit investigation of varied limb segment lengths in the tinamou model, so as to explore birds with disparate intralimb proportions, was well beyond the scope of this study, and would have required re-tuning of MTU architectural parameters for each morphotype tested (e.g., Modenese et al. 2016). Nevertheless, a rapid predictive simulation framework such as that outlined here makes this type of *in silico* investigation possible, which is a worthwhile avenue of future inquiry. Pending the undertaking of such a study, the above finding warrants re-assessment of predatory ecology in certain extinct carnivores. For instance, jumping onto large prey has been both scientifically inferred and popularized as a key predatory behavior in dromaeosaurid dinosaurs (Paul 1988; Ostrom 1994; Manning et al. 2006). Yet, derived eudromaeosaurids such as *Deinonychus* and *Velociraptor* had apomorphically foreshortened, robust metatarsi compared with more basal dromaeosaurids and other similarly-sized non-avian theropods (Ostrom 1976; Gatesy and Middleton 1997; Turner et al. 2012; Dececchi et al. 2020). This suggests that these forms probably had poorer jumping ability compared with other similarly-sized theropods, supporting inferences of predation upon animals that were generally smaller than their own body size (Fowler et al. 2011; Bishop 2019). Future studies can explicitly test this possibility using the modeling approach outlined here.

## Conclusion

By synthesizing a sophisticated musculoskeletal model with state-of-the-art numerical methods in optimal control, vertical jumping performance in a bird has been simulated for the first time. Based on physical principles alone, simulations were able to capture the salient components of jumping mechanics in birds, including a countermovement during launch, representing broadly successful simulation outcomes despite limited empirical data for direct validation. However, lowered whole-animal and muscle–tendon performance reveals that some aspects remain to be correctly emulated, such as mechanisms that increase peak MTU power. Additionally, only a single species of bird has been investigated. Nonetheless, it seems possible that generalized species that lack anatomical adaptations for jumping may rely more on mechanical principles, rather than those implicit to muscle–tendon function, to improve jump performance, although this could vary with animal size. This encouraging result provides the foundation for exploring jumping performance in other species of biped, extant and extinct.

## Supplementary Material

obab006_Supplementary_Data
