# Supplementary material for: Predictive Simulations of Musculoskeletal Function and Jumping Performance in a Generalized Bird
Source: Integr Org Biol. 2021 Apr 15;3(1):obab006. doi: 10.1093/iob/obab006 (PMC8341896; doi:10.1093/iob/obab006)
Supplement: obab006_Supplementary_Data [file obab006_supplementary_data.zip › French Abstract.docx]

**Simulations prédictives de la fonction musculo-squelettique et des performances de saut chez un oiseau généralisé**

Sauter est un comportement commun, mais exigeant, que de nombreux animaux utilisent au cours de leurs activités quotidiennes. Contrairement aux spécialistes du saut tels que les anoures et certains primates, la biomécanique du saut et les facteurs qui influencent la performance restent peu étudiés pour les espèces généralisées qui n'ont pas d'adaptations marquées pour le saut. Les approches de modélisation biomécanique computationnelle offrent un moyen d'aborder cette question de manière rigoureuse et mécaniste. Ici, la théorie du contrôle optimal et la modélisation musculo-squelettique sont intégrées pour générer des simulations prédictives du saut en hauteur maximal chez un petit oiseau terrestre, le tinamou. Un modèle musculo-squelettique tridimensionnel avec 36 actionneurs par patte est utilisé, et une méthode numérique nommée "direct collocation" est employée pour formuler un problème de contrôle optimal rapidement résoluble impliquant les phases de décollage et d'atterrissage. La simulation qui en résulte élève le centre de masse du corps entier à plus du double de sa hauteur debout, et les aspects clés du comportement simulé reproduisent qualitativement les observations empiriques d'autres oiseaux sauteurs. Cependant, les performances quantitatives sont moindres, avec une réduction des forces au sol, des hauteurs de saut et de la puissance musculo-tendineuse. Une manœuvre de contre-mouvement prononcée est utilisée pendant le lancement. Il a été démontré que l'utilisation d'un contre-mouvement est essentielle à l'obtention de hauteurs de saut plus importantes, et il se peut que ce phénomène doive exploiter uniquement des principes physiques pour réussir ; l'amplification de la performance musculaire n'est pas nécessairement une raison immédiate de l'utilisation de cette manœuvre. L'augmentation de la force musculaire ou de la vitesse de contraction au-dessus des valeurs nominales améliore grandement la performance de saut et, fait intéressant, a le plus grand effet sur les muscles extenseurs des membres plus distaux (c'est-à-dire ceux de la cheville), ce qui suggère que le membre distal peut être un lien critique pour le comportement de saut. Ces résultats justifient une réévaluation des déductions précédentes de la capacité de sauter chez certaines espèces éteintes avec des segments de membres distaux raccourcis, comme les dinosaures droméosauridés.
