# Supplementary material for: Predictive Simulations of Musculoskeletal Function and Jumping Performance in a Generalized Bird
Source: Integr Org Biol. 2021 Apr 15;3(1):obab006. doi: 10.1093/iob/obab006 (PMC8341896; doi:10.1093/iob/obab006)
Supplement: obab006_Supplementary_Data [file obab006_supplementary_data.zip › German Abstract.docx]

**Prädiktive Simulationen der muskuloskelettalen Funktion und Sprungleistung bei einem generalisierten Vogel**

Springen ist ein übliches jedoch anstrengendes Verhalten, das viele Tiere bei ihren täglichen Aktivitäten einsetzen. Im Gegensatz zu Springspezialisten, wie Fröschen und einigen Primaten, sind bei allgemeinen Arten, welche keine ausgeprägten Anpassung für Sprungverhalten aufweisen, die Biomechanik beim Springen und die Faktoren, welche die Leistungsfähigkeit beeinflussen, noch wenig untersucht. Computergestützte biomechanische Modellierungsverfahren bieten hier eine Möglichkeit, dies in einer gründlichen, mechanistischen Weise anzugehen. In dieser Arbeit werden die optimale Steuerungstheorie und Muskel-Skelett-Modellierung zusammen eingesetzt, um die maximale Sprunghöhe eines kleinen bodenlebenden Vogels, eines Perlsteisshuhns, zu simulieren und zu prognostizieren. Es wird ein dreidimensionales Muskel-Skelett-Modell mit 36 Aktuatoren pro Bein verwendet, und durch direkte Kollokation wird ein schnell lösbares optimales Steuerungsproblem formuliert, das sowohl die Abstoss- als auch die Landephase umfasst. Die daraus folgende Simulation bringt den Ganzkörperschwerpunkt auf mehr als das Doppelte seiner Standhöhe und entscheidende Aspekte des simulierten Verhaltens entsprechen qualitativ empirischen Beobachtungen für andere springende Vögel. Allerdings ist die quantitative Leistungsfähigkeit geringer, mit reduzierten Bodenkräften, Sprunghöhen und Muskel-Sehnen-Kräften. Beim Abstossen wird ein ausgeprägtes Gegenbewegungsmanöver durchgeführt. Die Durchführung einer Gegenbewegung ist nachweislich entscheidend für das Erreichen grösserer Sprunghöhen, wobei dieses Phänomen möglicherweise nur physikalische Prinzipien auszuschöpfen braucht, um erfolgreich zu sein. Die Verstärkung der Muskelleistung ist daher möglicherweise nicht zwingend ein unmittelbarer Grund für die Verwendung dieses Manövers. Eine Erhöhung der Muskelkraft oder der Kontraktionsgeschwindigkeit über die Nominalwerte hinaus führt zu einer erheblichen Zunahme der Sprungleistung und hat interessanterweise den grössten Effekt bei den weiter distal gelegenen Streckmuskeln der Beine (d.h. bei denjenigen des Sprunggelenks), was darauf hindeutet, dass die distale Gliedmasse ein entscheidendes Element für das Sprungverhalten sein könnte. Diese Ergebnisse geben Anlass zur Überprüfung früherer Schlussfolgerungen hinsichtlich der Sprungfähigkeit einiger ausgestorbener Arten mit verkürzten distalen Gliedmassen, wie beispielsweise bei dromaeosauriden Dinosauriern.
