# Supplementary material for: Predictive Simulations of Musculoskeletal Function and Jumping Performance in a Generalized Bird
Source: Integr Org Biol. 2021 Apr 15;3(1):obab006. doi: 10.1093/iob/obab006 (PMC8341896; doi:10.1093/iob/obab006)
Supplement: obab006_Supplementary_Data [file obab006_supplementary_data.zip › Revised Supplementary Material_ammendedPJB.pdf]

Supplementary Material for

# Predictive simulations of musculoskeletal function and jumping performance in a generalized ground bird

P.J. Bishop[1,2,*], A. Falisse[3,4], F. De Groote[3] and J.R. Hutchinson[1]

[1]Structure and Motion Laboratory, Department of Comparative Biomedical Sciences, Royal Veterinary College, Hatfield, United Kingdom
[2]Geosciences Program, Queensland Museum, Brisbane, Australia
[3]Department of Movement Sciences, KU Leuven, Leuven, Belgium
[4]Department of Bioengineering, Stanford University, Stanford, CA 94305, USA.

*Current address: Museum of Comparative Zoology, Department of Organismic and Evolutionary Biology, Harvard University, Cambridge, MA 02138, USA; pbishop@fas.harvard.edu.

Contents:

1. Torque-driven simulation
2. Supplementary figures
3. Supplementary table
4. Captions for supplementary movies
5. References

## 1. Torque-driven simulation

The original muscle-driven optimal control problem (OCP) described in the main text was re-formulated such that the model was driven by torque motors rather than muscle–tendon units (MTUs). All MTUs were removed from the OCP, and each degree of freedom in the hindlimb was appended by a torque actuator. The maximal output for each actuator was set equal to the maximal absolute external joint moment in the nominal muscle-driven simulation. Additionally, to improve simulation smoothness, a first-order differential equation was used to describe time-delayed motor activation (Falisse et al. 2019). The objective function in this modified OCP was as follows:

$$J = \int_{t_0}^{t_{\text{final}}} \begin{pmatrix} w_1 \sum_{l=1}^{L} a_l^2 + \\ w_2 \sum_{l=1}^{L} T_l^2 + \\ w_{3,\text{pelvis}} \sum_{p=1}^{P} \ddot{q}_p^2 + w_{3,\text{leg}} \sum_{l=1}^{L} \ddot{q}_l^2 + \end{pmatrix} dt - w_5 \cdot h_{\text{COM}}, \quad (1)$$

which aimed to minimize four terms:

1. The sum of squared torque motor activations ($a_l$) across all $L$ DOFs in the leg (= 6 in the current model), integrated across the simulation.
2. The sum of squared passive joint moments ($T_l$) across all $L$ DOFs in the leg, integrated across the simulation.
3. The sum of squared pelvis ($\ddot{q}_p$) and leg ($\ddot{q}_l$) coordinate accelerations across all $P$ and $L$ DOFs in the pelvis and leg, respectively (= 3 and 6 in the current model), integrated across the simulation.
4. The negative of the height reached by the whole-body COM ($h_{\text{COM}}$) at the mid-point of the simulation.

All other aspects of the OCP, including symmetry, path constraints, initial guess, bounds, weighting terms and scaling factors, were kept identical to the muscle-driven OCP.

## 2. Supplementary figures

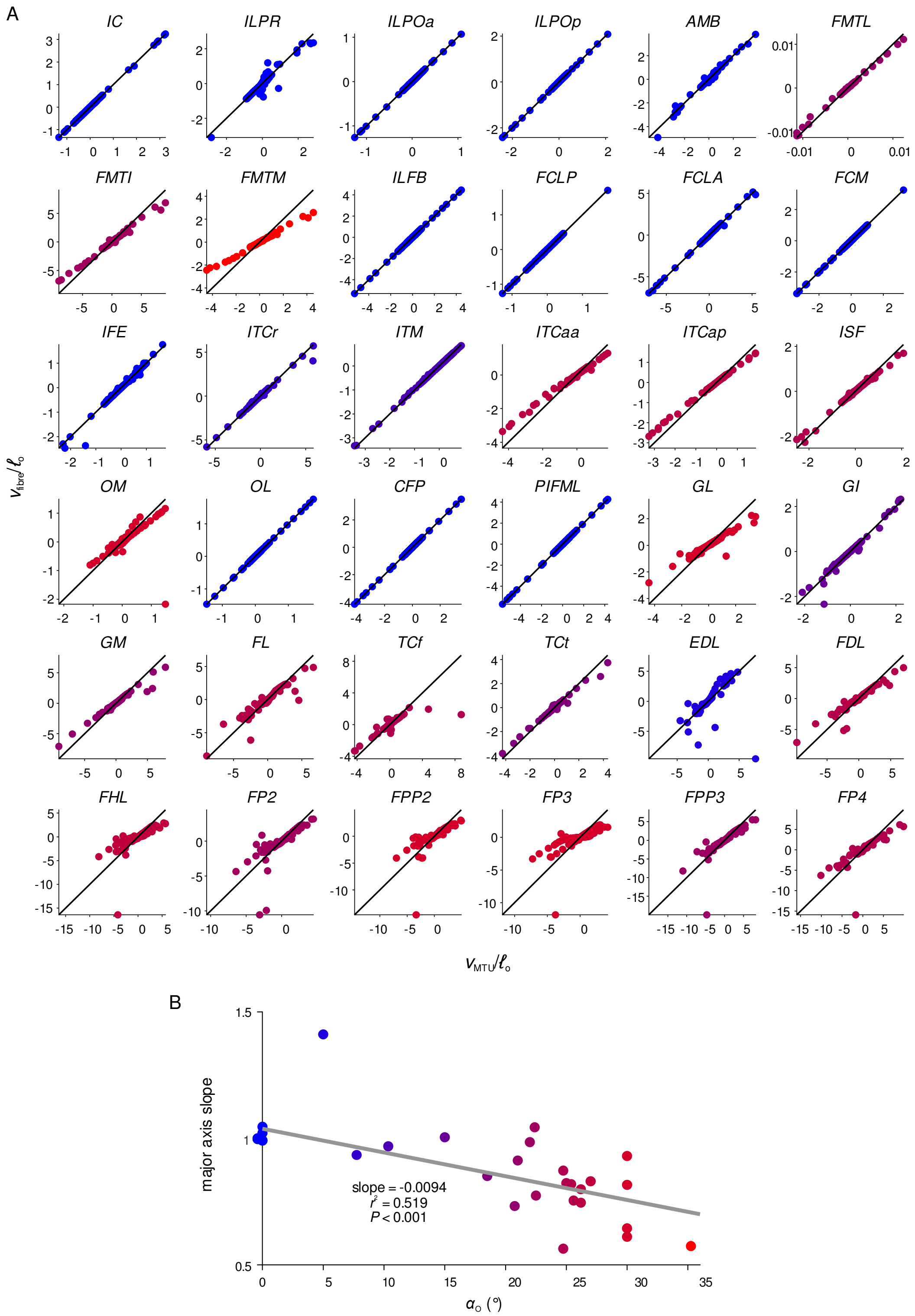


**Fig. S1.** (A) Comparison of fibre velocity and MTU velocity for all MTUs, both normalized by fibre length, in the nominal simulation; also plotted is the line of parity. See Table S1 for abbreviations. (B) A major axis regression was fitted to each plot in (A), and the resulting slope was compared to pennation angle via ordinary least squares regression in PAST 3.09 (Hammer et al. 2001). This revealed a significant relationship, whereby higher pennation angles (redder colours in both panels) tend to lead to slopes more different from one (lines of parity in A); therefore, change in fibre length is more decoupled from change in MTU length with higher pennation angles.

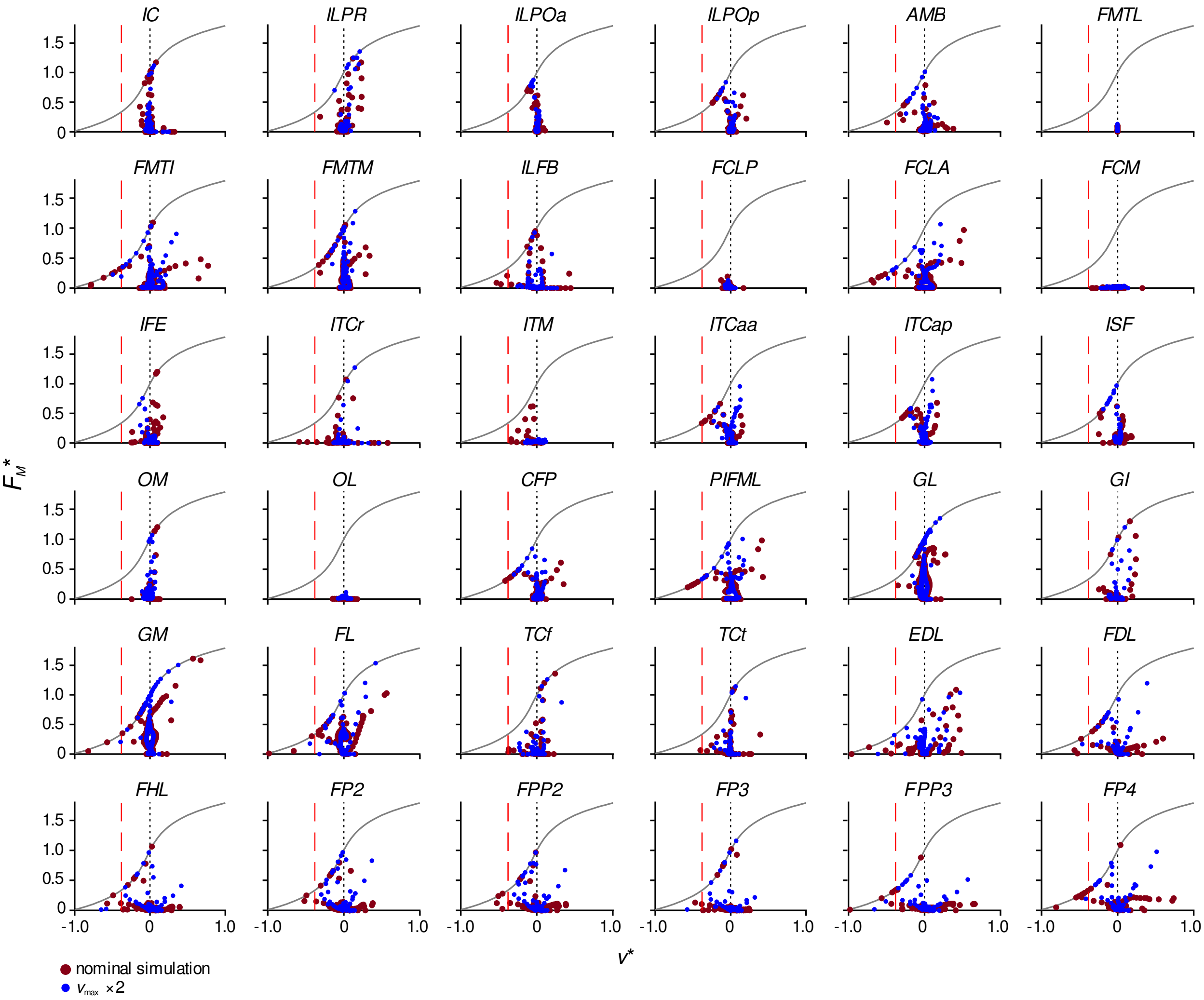


**Fig. S2.** Comparison of the muscle fibre component of MTU force normalized by maximum isometric force ($F_M$*) *versus* fibre velocity normalized by maximum contraction velocity ($v$*), for both the nominal simulation (maroon) and the sensitivity analysis where maximum contraction velocity was doubled (blue). Also indicated in grey is the fibre force–velocity curve (De Groote et al. 2016), and in red is the normalized contraction velocity at which fibre power is maximized (= 0.38×$v_{max}$). Variation along the vertical axis reflects differences in activation levels; points on the force–velocity curve indicate maximal MTU activation. Note how fibre velocity in the double-speed simulation tends to remain more isometric (closer to zero), indicating that absolute fibre velocities were not proportionally increased upon those of the nominal simulation. See Table S1 for abbreviations.

## 3. Supplementary table

**Table S1.** Muscle–tendon units included in the musculoskeletal model, with architectural parameters as derived previously (Bishop et al. 2021). The mass and maximal isometric force for the ILPO and ITC was split evenly between two actuators. Superscripts indicate about which major joint the MTU acts as an extensor (H = hip, K = knee, A = ankle).

| | Abbreviation | Belly mass, m (g) | Fibre length, $\ell_o$ (mm) | Tendon slack length, $L_S$ (mm) | Pennation angle, $\alpha_o$ (°) | Maximal isometric force, $F_{max}$ (N) |
|---|---|---|---|---|---|---|
| Iliotibialis cranialis[K] | IC | 2.49 | 70.31 | 3.09 | 0 | 11.324 |
| Iliotibialis lateralis pars preactabularis[K] | ILPR | 0.521 | 31.16 | 45.15 | 0 | 5.118 |
| Iliotibialis lateralis pars postacetabularis (anterior part)[H] | ILPOa | 2.15 | 69.85 | 2.71 | 0 | 9.814 |
| Iliotibialis lateralis pars postacetabularis (posterior part)[H] | ILPOp | 2.15 | 74.88 | 1.31 | 0 | 9.814 |
| Ambiens[K] | AMB | 0.3 | 23.05 | 28.57 | 0 | 3.952 |
| Femorotibialis lateralis[K] | FMTL | 1.565 | 37.07 | 0.61 | 21 | 14.913 |
| Femorotibialis intermedius[K] | FMTI | 0.449 | 14.73 | 32.45 | 22.5 | 9.33 |
| Femorotibialis medialis[K] | FMTM | 2.09 | 31.02 | 15.5 | 35.25 | 18.845 |
| Iliofibularis[H] | ILFB | 2.133 | 70.95 | 0.7 | 0 | 8.263 |
| Flexor cruris lateralis pars pelvica[H] | FCLP | 4.52 | 77.37 | 4.21 | 0 | 20.101 |
| Flexor cruris lateralis pars accessoria[H] | FCLA | 0.844 | 39.93 | 30.37 | 0 | 7.276 |
| Flexor cruris medialis[H] | FCM | 0.247 | 41.25 | 16.8 | 0 | 1.907 |
| Iliofemoralis externus | IFE | 0.212 | 11.34 | 5.96 | 0 | 6.098 |
| Iliotrochantericus cranialis | ITCr | 0.325 | 15.82 | 13.05 | 7.75 | 6.378 |
| Iliotrochantericus medius | ITM | 0.066 | 12.89 | 3.33 | 10.35 | 1.86 |
| Iliotrochantericus caudalis (anterior part) | ITCaa | 1.35 | 19.07 | 16.13 | 26.2 | 24.65 |
| Iliotrochantericus caudalis (posterior part) | ITCap | 1.35 | 23.15 | 1.06 | 26.2 | 24.65 |
| Ischiofemoralis[H] | ISF | 0.56 | 15.53 | 15.78 | 24.75 | 11.114 |
| Obturatorius medialis | OM | 0.619 | 13.55 | 22.38 | 30 | 14.655 |
| Obturatorius lateralis | OL | 0.0320 | 14.72 | 0.2 | 0 | 0.624 |
| Caudofemoralis pars pelvica[H] | CFP | 1.009 | 32.77 | 0.2 | 0 | 10.687 |
| Puboischiofemoralis medialis et lateralis[H] | PIFML | 1.543 | 38.53 | 0.3 | 0 | 12.134 |
| Gastrocnemius pars lateralis[A] | GL | 2.55 | 26.63 | 68.33 | 30 | 29.816 |
| Gastrocnemius pars intermedia[A] | GI | 0.438 | 39.29 | 53.54 | 15 | 3.425 |
| Gastrocnemius pars medialis[A] | GM | 2.251 | 27.44 | 64.24 | 20.75 | 25.305 |
| Fibularis longus[A] | FL | 2.315 | 28.22 | 118.37 | 25.4 | 24.198 |

| | | | | | | |
|---|---|---|---|---|---|---|
| Tibialis cranialis caput femorale[K] | TCf | 0.519 | 16.95 | 67.28 | 24.75 | 9.415 |
| Tibialis cranialis caput tibiale | TCt | 0.979 | 37.32 | 38.94 | 18.5 | 9.049 |
| Extensor digitorum longus | EDL | 0.295 | 17.88 | 112.59 | 5 | 4.688 |
| Flexor digitorum longus[A] | FDL | 0.563 | 27.41 | 104.71 | 25.6 | 5.963 |
| Flexor hallucis longus[A] | FHL | 0.304 | 16.87 | 143.74 | 27 | 5.124 |
| Flexor perforatus digitorum II[A] | FP2 | 0.524 | 20.57 | 124.43 | 22.4 | 7.277 |
| Flexor perforans et perforatus digitorum II[A] | FPP2 | 0.369 | 19.63 | 130.56 | 30 | 5.378 |
| Flexor perforatus digitorum III[A] | FP3 | 0.264 | 16.26 | 138.97 | 30 | 4.615 |
| Flexor perforans et perforatus digitorum III[A] | FPP3 | 0.214 | 17.05 | 140.82 | 22 | 3.569 |
| Flexor perforatus digitorum IV[A] | FP4 | 0.263 | 19.42 | 125.4 | 25 | 3.879 |

## 4. Captions for supplementary movies

**Movie S1.** Animation of the nominal muscle-driven jumping simulation, showing kinematics, ground reaction forces and muscle activation patterns, played at 0.25× real speed. Muscles become redder as they become more activated. Also shown is the location of the instantaneous whole-body centre of mass.

**Movie S2.** Animation of jumping simulations resulting from the sensitivity analyses, played at 0.5× real speed, comparing each variant to the nominal simulation. Also shown is the location of the instantaneous whole-body centre of mass for each model.

**Movie S3.** Animation of the nominal torque-driven jumping simulation, showing kinematics and ground reaction forces, played at 0.25× real speed. Also shown is the location of the instantaneous whole-body centre of mass.

## 5. References

Bishop PJ, Michel KB, Falisse A, Cuff AR, Allen VR, De Groote F, Hutchinson JR. 2021. Computational modelling of muscle fibre operating ranges in the hindlimb of a small ground bird (*Eudromia elegans*), with implications for modelling locomotion in extinct species. PLOS Computational Biology 17:1008843.

De Groote F, Kinney AL, Rao AV, Fregly BJ. 2016. Evaluation of direct collocation optimal control problem formulations for solving the muscle redundancy problem. Annals of Biomedical Engineering. 44:2922-2936.

Falisse A, Serrancolí G, Dembia CL, Gillis J, Jonkers I, De Groote F. 2019. Rapid predictive simulations with complex musculoskeletal models suggest that diverse healthy and pathological human gaits can emerge from similar control strategies. Journal of the Royal Society Interface. 16:20190402.

Hammer Ø, Harper DAT, and Ryan PD. 2001. Past: Paleontological statistics software package for education and data analysis. Palaeontologia Electronica. 4:4.
