# Supplementary material for: Predictive Simulations of Musculoskeletal Function and Jumping Performance in a Generalized Bird
Source: Integr Org Biol. 2021 Apr 15;3(1):obab006. doi: 10.1093/iob/obab006 (PMC8341896; doi:10.1093/iob/obab006)
Supplement: obab006_Supplementary_Data [file obab006_supplementary_data.zip › Cover Image Caption.doc]

Predictive simulation of vertical jumping in a small bird, the elegant-crested tinamou, using musculoskeletal modelling and dynamic optimization methods. The muscles become more heavily recruited (glow redder) as the bird pushes off the ground to become airborne. Image credit: P.J. Bishop.
