# Supplementary material for: Predictive Simulations of Musculoskeletal Function and Jumping Performance in a Generalized Bird
Source: Integr Org Biol. 2021 Apr 15;3(1):obab006. doi: 10.1093/iob/obab006 (PMC8341896; doi:10.1093/iob/obab006)
Supplement: obab006_Supplementary_Data [file obab006_supplementary_data.zip › Dutch Abstract.doc]

**Voorspellende simulaties van musculoskeletale functie en springprestaties bij een gegeneraliseerde vogel**

Springen is een veel voorkomend, maar veeleisend, gedrag dat veel dieren toepassen tijdens hun dagelijkse bezigheden. In tegenstelling tot de springspecialisten zoals de anura en sommige primaten, is de biomechanica van het springen en de factoren die de prestaties beïnvloeden nog weinig bestudeerd voor algemene soorten die geen uitgesproken adaptaties voor het springen hebben. Computationele biomechanische modelbenaderingen bieden een manier om dit op een rigoureuze, mechanistische manier aan te pakken. Hier worden optimale controle theorie en musculoskeletale modellering geïntegreerd om voorspellende simulaties te genereren van maximale hoogtesprong bij een kleine grondbewonende vogel, een tinamou. Een driedimensionaal musculoskeletaal model met 36 actuatoren per poot wordt gebruikt, en directe collocatie wordt toegepast om een snel oplosbaar optimaal controleprobleem te formuleren dat zowel de opstijg- als de landingsfase omvat. De resulterende simulatie verhoogt het lichaamszwaartepunt tot meer dan het dubbele van de stahoogte, en belangrijke aspecten van het gesimuleerde gedrag komen kwalitatief overeen met empirische waarnemingen voor andere springende vogels. De kwantitatieve prestaties zijn echter minder, met verminderde grondkrachten, spronghoogtes en spierpeeskracht. Tijdens de lancering wordt een uitgesproken tegenbewegingsmanoeuvre gebruikt. Aangetoond is dat het gebruik van een tegenbeweging van cruciaal belang is voor het bereiken van grotere spronghoogten, en dit fenomeen hoeft alleen op fysische principes te berusten om succesvol te zijn; versterking van de spierprestaties hoeft niet noodzakelijk een proximate reden te zijn voor het gebruik van deze manoeuvre. Het verhogen van de spierkracht of van de contractiesnelheid boven de nominale waarden verbetert de sprongprestatie aanzienlijk, en heeft interessant genoeg het grootste effect op de meer distale extensoren van de ledematen (d.w.z. die van de enkel), wat suggereert dat de distale ledematen een kritieke schakel kunnen zijn voor het springgedrag. Deze resultaten rechtvaardigen een herevaluatie van eerdere conclusies over springvermogen bij sommige uitgestorven soorten met voorgekorte distale ledematen, zoals dromaeosauride dinosauriërs.
